# Deep learning for detecting and characterizing oil and gas well pads in satellite imagery

**DOI:** 10.1038/s41467-024-50334-9

**Published:** 2024-08-15

**Authors:** Neel Ramachandran, Jeremy Irvin, Mark Omara, Ritesh Gautam, Kelsey Meisenhelder, Erfan Rostami, Hao Sheng, Andrew Y. Ng, Robert B. Jackson

**Affiliations:** 1 Stanford Research Computing, Stanford University, Stanford, CA USA; 2 Department of Earth System Science, Stanford University, Stanford, CA USA; 3 Department of Computer Science, Stanford University, Stanford, CA USA; 4 Environmental Defense Fund, Austin, TX USA; 5 Woods Institute for the Environment and Precourt Institute for Energy, Stanford University, Stanford, CA USA

**Keywords:** Climate-change mitigation, Attribution

## Abstract

Methane emissions from the oil and gas sector are a large contributor to climate change. Robust emission quantification and source attribution are needed for mitigating methane emissions, requiring a transparent, comprehensive, and accurate geospatial database of oil and gas infrastructure. Realizing such a database is hindered by data gaps nationally and globally. To fill these gaps, we present a deep learning approach on freely available, high-resolution satellite imagery for automatically mapping well pads and storage tanks. We validate the results in the Permian and Denver-Julesburg basins, two high-producing basins in the United States. Our approach achieves high performance on expert-curated datasets of well pads (Precision = 0.955, Recall = 0.904) and storage tanks (Precision = 0.962, Recall = 0.968). When deployed across the entire basins, the approach captures a majority of well pads in existing datasets (79.5%) and detects a substantial number (>70,000) of well pads not present in those datasets. Furthermore, we detect storage tanks (>169,000) on well pads, which were not mapped in existing datasets. We identify remaining challenges with the approach, which, when solved, should enable a globally scalable and public framework for mapping well pads, storage tanks, and other oil and gas infrastructure.

## Abstract (web summary)

This work uses deep learning on satellite imagery to map well pads and storage tanks in two major U.S. basins. The resulting data fills large gaps in existing databases, a crucial step for improving methane emission estimates and source attribution.

## Introduction

Methane emissions are a key contributor to climate warming, accounting for around 30% of the recent rise in global temperatures^1^. Methane absorbs radiation more efficiently than CO_2_; the pound-for-pound Global Warming Potential (GWP) of methane is ~83 times greater than CO_2_ over a 20-year time period, and ~30 times greater over a 100-year time period^2^. Methane’s atmospheric lifetime is about a decade and significantly shorter than that of carbon dioxide. Consequently, mitigating methane emissions provides one of the best opportunities for reducing the rate of warming over the next decade or two.

Substantial rises in methane emissions over the past four decades, and in particular over the last 15 years, can be attributed primarily to anthropogenic factors, of which fossil fuels are a leading contributor (35% of anthropogenic methane emissions)^3–5^. As such, mitigation measures to reduce methane emissions from the fossil fuel sector are considered to be among the most attractive and cost-effective options available^5–8^. A large portion of methane emissions from the oil and gas (O&G) industry arise during production, specifically from O&G infrastructure such as well pads^9^. Moreover, aerial surveys suggest that most large sources of methane from well pads emanate from storage tanks^10^. Therefore the detection, quantification, and mitigation of methane emissions from well pads and storage tanks remain high priorities and motivate the work presented here.

A critical component of both measuring and mitigating methane emissions in the O&G sector is the availability of transparent, comprehensive, and accurate geospatial information for O&G facilities. Such a repository would enable more accurate bottom-up (BU) estimates of methane emissions and granular source attribution from top-down (TD) estimates, including those from satellites. BU estimates of methane emissions have been frequently observed to underestimate overall emissions, in part attributable to uncertainties in activity (i.e., count) data in terms of the underlying inventory of facilities and equipment^11,12^. Meanwhile, advancements in aerial-based and satellite-based observations have improved the TD quantification of methane emissions. Several satellite missions over the past decade have provided useful information on methane emissions globally; this was not possible with conventional ground measurements and airborne campaigns of limited spatial context^13^. Global satellites (e.g., SCIAMACHY, GOSAT, TROPOMI) can detect coarse methane “hot spots”, whereas other satellites and airborne survey instruments (GHGSat, AVIRIS-NG) can detect high emission sources at a fine-grained resolution, albeit with more limited spatial coverage^14^. MethaneSAT and CarbonMapper, satellites launching in 2024, allow methane emissions to be tracked with relatively high resolution and broad spatial coverage, enabling improved quantification of area emissions and high-emitting point sources. Performing both area-source and point-source attribution on such top-down obtained methane emissions data requires the comprehensive dataset of methane-emitting O&G infrastructure mentioned above.

Reconciling the disagreement between BU/TD emission estimates and performing accurate methane source attribution have been shown to be successful in limited geographic areas such as the Barnett shale and California thanks to granular, facility-level O&G databases manually assembled and validated from existing public data sources and/or remote sensing imagery^11,15^. However, large data gaps exist both nationally and globally, and extending these manual approaches is infeasible at larger scales. In the United States, the Department of Homeland Security’s Homeland Infrastructure Foundation-Level Data (HIFLD) program^16^ aggregates state-level well data into a national database. The sources of this data include required reporting by facility operators and publicly available state data, which can be outdated and vary in scope and coverage from state to state^17^. Uncertainty about the total number of active wells exists even between agencies like the EPA and EIA, which cite different national totals^17^. This uncertainty is compounded by abandoned and orphaned wells, which are often undocumented. Furthermore, sub-facility inventories of equipment such as storage tanks, which are significant sources of production-site methane emissions^10,18^, are scarce. At the global level, recently there has been a comprehensive development of a spatially explicit oil and gas infrastructure database using bottom-up reported data; however, major gaps exist in terms of well pad and storage tank data which are readily not available in bottom-up sources globally^19^.

The emergence of freely available, high-resolution remote sensing imagery, coupled with recent progress in deep learning methods, presents a promising opportunity for filling many of these data gaps and updating them regularly. Deep learning techniques have been increasingly applied to fine-scale infrastructure mapping efforts, from building footprint detection^20^ and urban land use classification^21^ to energy infrastructure identification^22^, including solar photovoltaics^23^, wind turbines^24^, oil refineries^25^, and other methane-emitting infrastructure^26^. The application of similar methods to methane-emitting O&G facilities and sub-facilities such as well pads and storage tanks to develop large-scale, granular, and accurate geospatial databases has the potential to address these challenges in emissions estimation and source attribution. Previous work has illustrated the potential for deep learning to effectively detect O&G well pads and storage tanks in the Denver-Julesburg (hereafter referred to as Denver) basin^27^ but the model was only manually assessed in small subregions of the basin, and not assessed against existing well pad data repositories. Here, we design a larger-scale framework, trained and validated on diverse facilities in multiple full basins.

In this work, we develop and deploy deep learning models to detect O&G well pads and storage tanks across the Permian and Denver basins automatically. We focus on these basins because of their high production rates (over 40% of U.S. oil production occurs in the Permian basin alone^28^) and high leakage rates (60% higher than the national average in the Permian basin^29^). These two basins also have diverse well-pad types and settings (including relatively arid landscapes in the Permian and dense urban landscapes in the broader Denver area), which are useful for testing the models across varied environments.

Our main contributions are as follows: First, we develop deep-learning approaches using public satellite imagery to map well pads and storage tanks. To do this, we carefully curate labeled datasets of satellite imagery with well pad and storage tanks annotations, and adapt well-established object detection models to perform well pad and storage tank detection by leveraging these datasets. Second, we present results from many experiments which rigorously evaluate the performance of the models, including several metrics capturing the proportion of false positive and false negatives in each basin, the impact of jointly training models in basins, the effect of well pad size on model performance, the benefits of using a well pad verification model for reducing false positives, and the generalization of the model to new regions. Crucially, both the well pad and storage tank detection approaches achieve high performance assessed against expert annotated data in the Permian and Denver basins. Third, we apply the final approach to create a database of well pads and storage tanks across the entire Permian and Denver basins, and conduct thorough analyses evaluating the quality of the data, including comparing to existing well pad databases. The deep learning-mapped well pads capture the majority of wells in existing databases that were visible in the satellite imagery and include a substantial number of well pads not captured in the existing datasets, illustrating the potential of these methods to fill data gaps. Through our analyses, we also identify remaining data and modeling challenges with the approach, which, if solved, would enable a globally scalable well pad and storage tank mapping framework.

## Results

### Training deep learning models to detect and verify well pads

We developed a well pad detection pipeline with two stages to detect and verify well pads in the Permian and Denver basins. In the first stage, we framed well pad detection as an object detection task, wherein a model was trained to input an image and output axis-aligned bounding boxes around instances of well pads. For this stage, we trained a RetinaNet^30^ detector with a ResNet-50^31^ backbone, and thresholded the model (hereafter referred to as the detection model) to maximize recall. In the second stage, we used a binary classifier to eliminate false positive detections and “verify” individual instances of well pads produced in the first stage by outputting whether or not a satellite image contains a well pad. We trained an EfficientNet-B3^32^ model, and thresholded the model (hereafter referred to as the verification model) to maximize precision. For training and validating both models, we collected a dataset of images of 88,044 images captured from the Google Earth^33^ satellite basemap, including 10,432 images with manually labeled well pads (positives) and 77,612 negative images. For evaluating the detection model, we used the terms “precision” (the proportion of model predictions that are actually well pads) and “recall” (the proportion of actual well pads in the dataset that were correctly identified by the model). We also used average precision (AP), which estimates the average precision across all recall thresholds and provides a more holistic way to compare model performance than precision and recall at a single threshold. The verification model is evaluated with precision and recall. We selected the best architecture, backbone, and hyperparameters for the detection and verification models based on which led to the highest performance on the validation set (see “Methods”, Supplementary Tables 1 and 2).

The full well pad detection pipeline demonstrated strong performance on a “held-out” test set comprising 10% of the overall dataset (Table 1). The model achieved high AP across all thresholds (0.959 in the Permian basin and 0.928 in the Denver basin). After choosing confidence thresholds to maximize recall in the detection stage and precision in the verification stage, the model achieved high overall precision (0.955) with over 0.9 recall. From inspecting a selection of the undetected well pads in both basins, we found that false negatives primarily occurred on small well pads, and partially visible well pads at the edge of images that do not meet our filtering criteria (described in the “Methods” section below). We note that while the latter issue affected evaluation metrics, we used overlapping tiles to prevent such errors during deployment. We found that false positives primarily occurred on facilities sharing visual similarity with well pads such as wind turbines, other O&G infrastructure (e.g., compressor stations), and other types of cleared land (e.g., cleared farmland).

**Table 1:** Test set results of the well pad detection pipeline for the Permian and Denver basins individually and together (“Overall”)

	Average precision (mean ± SD)	Precision (mean ± SD)	Recall (mean ± SD)
Permian	0.959 ± 0.002	0.975 ± 0.003	0.906 ± 0.005
Denver	0.928 ± 0.003	0.935 ± 0.005	0.901 ± 0.007
Overall	0.944 ± 0.002	0.955 ± 0.004	0.904 ± 0.006

Because positive examples were downloaded centered around well pads, we used stochastic data augmentations (random cropping and scaling) when evaluating the model to simulate the distribution of well pads during deployment to the full basins, where well pads are rarely centered in the image. As such, the results are reported as the mean and standard deviation (SD) across 10 runs.

We also examined the effect of the second stage of our detection pipeline, wherein the verification model eliminates false positives and “verifies” the remaining well pad detections. When evaluated independently, the verification model achieved high performance in both basins: On the test set, the models achieved perfect precision (1.0) in both basins while maintaining high recall (>0.97).

To validate the advantage of using the verification model, we compared the performance of the stand-alone detection model with the detection model in conjunction with the verification model. To evaluate the models together, we downloaded images centered at every candidate prediction produced by the detection model to feed to the verification model; candidates that were not “verified” were removed from the predictions. We then used precision and recall to evaluate the remaining “verified” predictions. We compared these results to the standalone detection model thresholded at the recall value obtained from evaluating the models together to determine if the verification model improved overall precision. The verification model improved precision by 0.013 and 0.009 on average in the Permian and Denver respectively, validating our hypothesis that a dedicated model for eliminating false positives by verifying candidate well pads is more effective than just using a higher confidence threshold with the standalone detection model (Supplementary Table 3).

When stratifying performance by basin, we found that the pipeline performs poorer in the Denver than the Permian basin. The AP was 0.031 lower in the Denver basin than in the Permian basin on average, indicating that over 3% more of the predictions were false positives on average across all confidence thresholds in the Denver basin. We mainly attribute the lower performance in the Denver basin to the higher prevalence of smaller well pads in the region, which the model identified less reliably than medium and large well pads in both basins (Table 2). We defined small well pads as <41 m^2^ (100 image pixels), large well pads as >164 m^2^ (400 image pixels), and medium well pads as those in between (Supplementary Fig. 1). We note that performance across medium and large well pads in both basins is roughly comparable, but that performance for small well pads is over 0.09 and 0.25 AP lower than medium and large-sized well pads in the Permian and Denver, respectively (small object detection remains a known challenge with current techniques^34^). Notably, only 5.2% of well pads in the Permian test set are designated as small, compared to 18.3% in the Denver basin (Table 2), where single wellhead pads are common. Thus, the overall performance in the Denver basin was relatively penalized by errors on small well pads. Further, the performance on small well pads is considerably worse in the Denver basin than in the Permian, a result we attribute to the lack of discernible features on small well pads in the Denver basin and the higher likelihood of incurring false positives (Supplementary Fig. 1). We also acknowledge that the Denver basin contains a broader range of “built” urban and suburban infrastructure than the Permian basin, which are also likely to contribute to false positives.

**Table 2:** Test set results of the detection pipeline stratified by well pad size

	Permian		Denver	
	% Well pads	Average precision (mean ± SD)	% Well pads	Average precision (mean ± SD)
Small (<41 m^2^)	5.2	0.853 ± 0.082	18.3	0.700 ± 0.011
Medium (41–164 m^2^)	83.7	0.962 ± 0.002	68.4	0.976 ± 0.005
Large (>164 m^2^)	11.1	0.944 ± 0.001	13.2	0.953 ± 0.012
Overall	–	0.959 ± 0.002	–	0.928 ± 0.003

The results are reported as the mean and standard deviation (SD) across 10 runs.

We experimented with training basin-specific detection models compared with training a detection model jointly in both basins and found that the jointly trained model outperformed both basin-specific models. The basin-specific models achieved the best performance when evaluated in the same basin they were trained in, but there was a significant drop in AP (>0.1) when the Permian model was evaluated in the Denver basin and vice versa, which is likely due to a large distribution shift between the regions. The model trained jointly in both basins outperformed the basin-specific models by 0.004 AP in the Permian basin and 0.006 AP in the Denver basin on average, indicating that despite the distribution shift, performance in both basins mutually benefited from jointly training the model (Supplementary Table 4). We thus selected the joint model for deployment in both regions.

Finally, we evaluated the model in new regions that were “unseen” during training to test the generalization of the model beyond the Permian and Denver basins. To do so, we collected additional labeled well pad datasets in the Appalachian, Texas-Louisiana-Mississippi (TX-LA-MS) Salt, Anadarko, and Uinta-Piceance basins, which together comprise over 38% of U.S. production^34^ and that span diverse geographies across the country (Supplementary Fig. 2). We collected an average of 4,774 images across each basin, including an average of 622 images with manually labeled well pads; we then evaluated our model trained solely in the Permian and Denver in these new basins. We found that performance varied considerably by basin: Notably, when evaluated in the Uinta-Piceance basin, the model achieved high precision and recall (0.948 and 0.943 respectively), exhibiting no decrease in performance as compared to the Permian and Denver basins. However, when evaluated in the Appalachian basin, the model achieved considerably lower precision and recall (0.647 and 0.552 respectively). Performance decreased slightly in the TX-LA-MS Salt (>0.8 precision and recall) and Anadarko (>0.85 precision and recall) basins but the model remained a relatively accurate detector of well pads. The results suggest that the model generalizes well in regions with low distribution shift; in the Uinta-Piceance basin, for example, well pads exhibit similar visual characteristics in an arid environment as in the Permian basin. Meanwhile, in the Appalachian basin, well pads are typically small with poorly defined footprints, and are often obscured by trees and shade. The model performance in the Appalachian indicates that the model is unable to generalize across a large distribution shift, and also that the well pad detection task may be significantly harder in some regions. Full results and dataset counts for the evaluation basins are shown in Supplementary Table 5 and satellite imagery examples are shown in and Supplementary Fig. 3.

### Basin-scale well pad deployment

We deployed our well pad detection pipeline to the entire Permian and Denver basins. We tiled both basins into >13.9 million images, spanning approximately 313,340 km^2^, that we evaluated for well pad and storage tank detections. Well pads were considered verified if they matched reported data from the Enverus^35^ and HIFLD Open Data^16^ datasets or if they were confirmed by the verification model; all unverified well pads were removed from the detections. The full deployment procedure is described in further detail in the Methods section. The deployment produced 194,973 well pad detections in the Permian and 36,591 detections in the Denver basin.

**Fig. 1:**
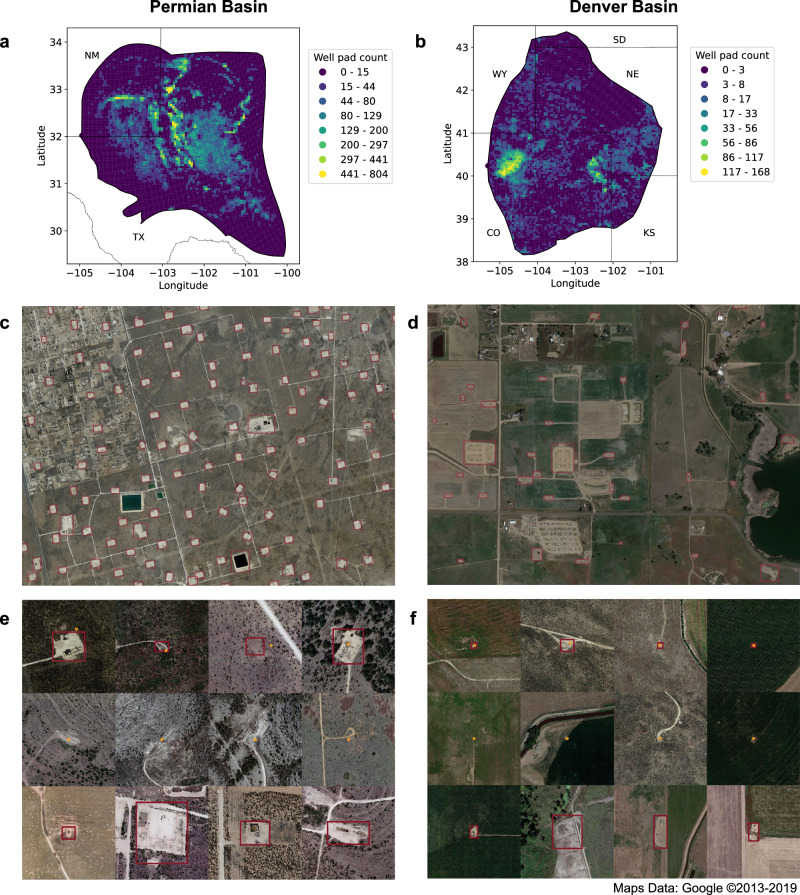
Visualization of basin-scale well pad deployments in the Permian and Denver basins. **a**, **b** Gridded density heatmaps of detected well pad counts. Each grid cell represents 5 km^2^. **c**, **d** Deployment detections (magenta boxes) from subregions of the basins. **e**, **f** Sample deployment detections (magenta boxes) of captured (first row), missed (second row), and new (third row) well pads. Deployment detections are matched against reported sources of data (displayed as orange circles).

We then analyzed our detections against reported data. We focused first on “captured” well pads (those present in our detections that matched reported data) and on “missed” well pads (those present in the reported data that were not detected by our model). We also analyzed “new” well pad detections (those that we detected but were not in the reported data, including actual well pads and false positives), plus the storage tank detections described in the next section. Sample detections and density heatmaps from the well pad deployment are shown in Fig. 1.

**Fig. 2:**
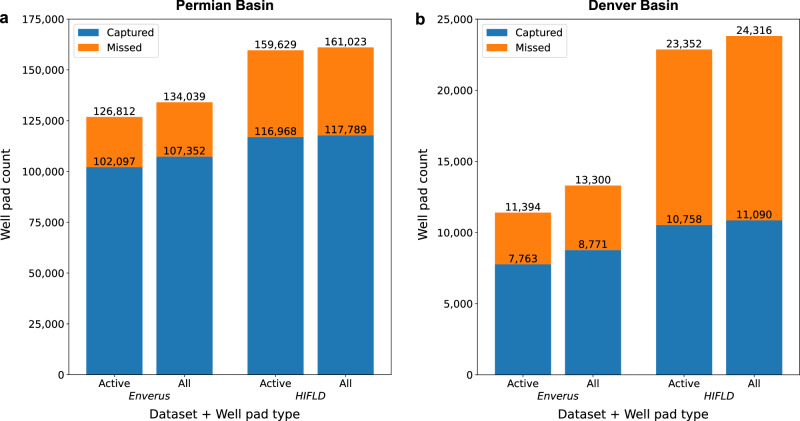
Deployment performance of the well pad detection pipeline assessed against reported well pad datasets. **a**, **b** We examined the recall rates of the well pad detection pipeline assessed against the Enverus^35^ and HIFLD Open Data^16^ well pad datasets for both active well pads in the reported data and against all well pads (active + inactive). Our methodology for classifying well pads as active/inactive relies mainly on leveraging the “Well Status” properties of the Enverus and HIFLD datasets (Supplementary Note 1).

For the Permian basin, we observed recall rates of 80.5% and 73.3% on active well pads when compared against the Enverus and HIFLD datasets, respectively. Similarly, in the Denver basin, we found recall rates of 68.1% and 46.1% when compared against the Enverus and HIFLD datasets (Fig. 2). We note that both datasets are not perfect sources to compare against as they may contain inaccurate coordinates and/or well pads that are no longer visible in satellite imagery (examples are shown in Supplementary Fig. 4); therefore, the recall rates calculated here likely underestimate the true proportion of well pads captured. Further, recall is considerably lower when comparing against the HIFLD dataset than against the Enverus dataset, and the number of reported well pads is also much higher in the former than the latter in both basins. We note that the HIFLD dataset was published in 2019 and contains aggregated state-level data which may be outdated in some states; the HIFLD data is only up-to-date as of 2014 in the Denver basin and 2018 in the Permian basin. Meanwhile, the Enverus dataset is frequently updated and is up-to-date as of 2021 in both basins. As such, the HIFLD dataset is more likely to contain decommissioned well pads that are no longer present in the Enverus dataset, which helps explain the disparity in recall and number of reported well pads in the datasets.

We found marginally lower recall rates on both datasets and in both basins when comparing against all well pads. Lower recall rates on the “all” category are attributable to the difficulty in detecting inactive well pads, which can contain little to no equipment, are rehabilitated by vegetation, or are no longer visible in imagery. Below, we focus mainly on assessing the detections against active well pads in the Enverus dataset for the reasons presented above, and because of access to site-level production data in the Enverus dataset, which are not available in the HIFLD dataset. Across both basins, the model detections capture 79.5% of active well pads in the Enverus dataset.

**Fig. 3:**
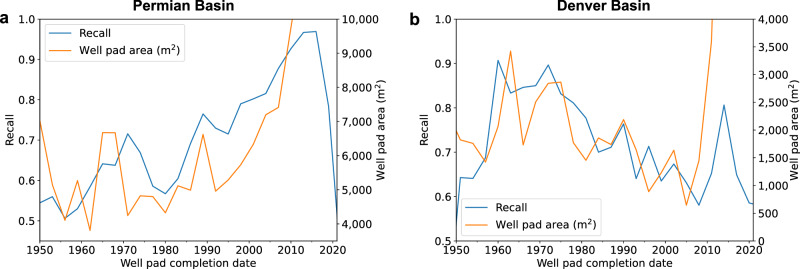
Comparison of deployment recall and well pad area (m^2^) over time. **a**, **b** Recall was assessed against active well pads in the Enverus dataset. Time was measured using the well pad construction completion date for well pads completed between 1950 and 2021, which comprise 99% of all well pads in the dataset.

**Fig. 4:**
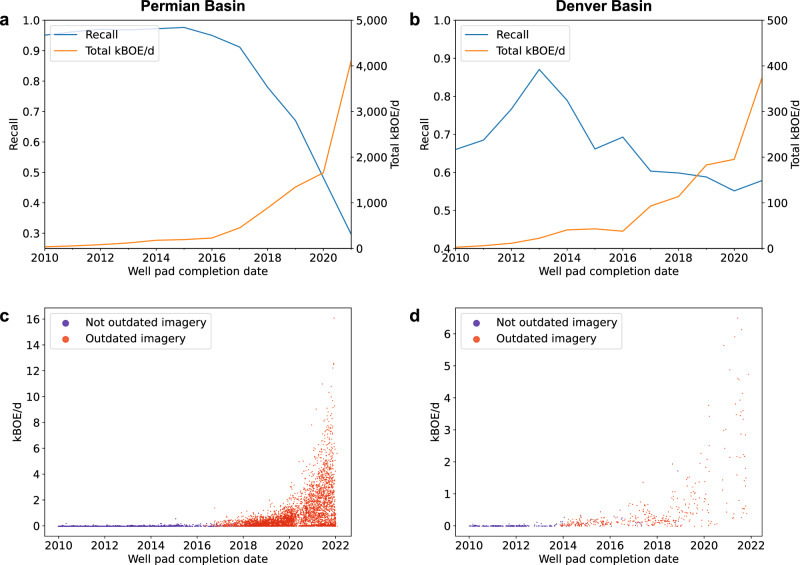
Analysis of recall, well pad production, and undetected well pads attributed to outdated imagery. **a**, **b** Comparison of deployment recall (assessed against active well pads in the Enverus dataset) and reported production over time, measured in kilo barrels of oil equivalent per day (kBOE/d). **c**, **d** Production of missed well pads over time. Each point represents a missed well pad and is color-coded based on whether the imagery is outdated in reference to undetected well pad (i.e., captured before its completion date) or not.

We also examined recall rates across time, and found that in the Permian basin, recall generally increased over time before dropping sharply in the mid-2010s. In the Denver basin, recall generally decreased over time before increasing and sharply decreasing in the 2010s. We found that recall rates largely correspond to the evolving size of constructed well pads, which we calculated based on the size of bounding box detections (Fig. 3). Recall is higher in years where larger well pads were constructed, and lower in years where smaller well pads were constructed. This trend is consistent with our observation that the model performs better on larger well pads (Table 2). However, well pad size does not explain the drop in performance on recently constructed well pads, as well pads are missed substantially while well pad size increases in the 2010s. In the Permian, recall remains high for well pads constructed from 2010 to 2016, then drops sharply from 2016 to 2021 (Fig. 4a). This trend is primarily due to outdated imagery in the Google Earth basemap, wherein recently constructed well pads may not appear in imagery acquired before their completion date. In the Permian, imagery acquisition dates range from 2014 to 2019 (Supplementary Table 6). For well pads completed in 2012 the recall rate is 0.97; these well pads are unaffected by outdated imagery. For well pads completed in 2019, the recall rate drops to 0.67, as 52.3% of imagery coverage in the basin is from 2018 or earlier and would not depict the well pad (Fig. 4a and Supplementary Table 6). We found a similar trend in the Denver basin.

Further compounding the impact of outdated imagery is the fact that recently constructed well pads account for a large majority of the production (measured in kilo barrels of oil equivalent per day, i.e. kBOE/d). In the Permian, the total kBOE/d produced by wells completed from 2016 to 2021 increases rapidly; these high-producing wells were largely missed by the model due to outdated imagery (Fig. 4a, c). The same trend is visible in the Denver basin. As such, outdated imagery has a disproportionate effect on production: In the Permian basin, well pad recall is 0.805, whereas production recall is only 0.555 (Table 3). Notably, by evaluating well pad detections against reported data while removing all reported well pads with outdated imagery, we saw a substantial increase in well pad recall, and nearly 100% production recall in the Permian basin, suggesting that outdated imagery is responsible for a large portion of recall issues on recently constructed wells at deployment. We found similar trends in the Denver basin (Table 3). Examples of the outdated satellite imagery are shown in Supplementary Fig. 4.

**Table 3:** Recall of well pads and production

		2010-2021		All Years	
		All well pads	Excluding outdated imagery well pads	All well pads	Excluding outdated imagery well pads
Permian recall	Well pads	0.889	0.966	0.805	0.833
	kBOE/d	0.541	0.992	0.555	0.981
Denver recall	Well pads	0.701	0.780	0.681	0.706
	kBOE/d	0.788	0.986	0.786	0.979

Rates are shown against all reported well pads (assessed against active well pads in the Enverus dataset), and excluding those with outdated Google imagery. Production is measured in kilo barrels of oil equivalent per day (kBOE/d).

We also found a large number of “new” well pad detections in both basins. Well pads were considered “new” if they were detected by the model but did not match any well pad, active or inactive, in the union of the Enverus and HIFLD datasets. 67,201 such detections were found in the Permian basin, and 24,525 in the Denver basin.

We evaluated a sample of 5000 new detections (*n* = 2500 in each basin), and found that 83.04% and 57.9% of the new detections were in fact well pads in the Permian and Denver basins respectively, while the remaining detections were false positives. These well pads represent new detections that were not previously recorded in available databases as of their published dates (2019 for HIFLD and 2021 for Enverus). Based on this sample, we estimate that our detection pipeline identified over 55,800 and 14,200 new well pads in the Permian and Denver basins respectively, a total increase of 33% over the existing repositories.

We note that 21.6% of new well pad detections in the sample were completely bare, i.e. containing no visible equipment such as pump jacks, storage tanks, well head fencing, or well heads (which may in some cases be too small to see in satellite imagery) typically used to discern well pads from other infrastructure. These “well pads” could represent plugged and abandoned/orphaned well pads, or well pad footprints that were cleared but not yet drilled at the time of imagery acquisition. As such, these “well pads” may not be expected to appear in reported well pad datasets. The remaining 78.4% did feature equipment, and yet also did not appear in the reported well-pad datasets. We also note that we distinguish the bare “well pads” from land cleared for other purposes (e.g. agriculture) through features such as proximity to other well pads, presence of characteristic road(s) leading to the site, and proximity to other infrastructure, which often indicate that a cleared area is not a well pad (i.e. a cleared region next to a farm is unlikely to be a well pad and more likely to be associated with agricultural use).

Based on the sample and the number of captured detections, we estimate the overall deployment precision to be 0.909, with estimated precisions of 0.944 and 0.720 in the Permian and Denver basins, respectively.

### Storage tank detection

In addition to the well pad detection pipeline, we developed a storage tank detection model, also framed as an object detection task, where the model outputs a bounding box for each storage tank on a well pad. We obtained 10,470 storage tank labels on 1833 well pad images, and used images of well pads without storage tanks as negatives to train a discriminative model. We selected a FasterRCNN^36^ architecture with a Res2Net^37^ backbone as the highest-performing model (see “Methods” section, Supplementary Table 7), evaluated with the same metrics as the well pad detection model (AP, precision, recall); we also used mean absolute error (MAE) to compare ground truth and predicted storage tank counts at the well pad level. We do not adopt the two-stage approach we used previously for detecting storage tanks because (a) the detection model achieves high precision and recall on its own and (b) verifying individual instances of storage tanks is difficult, as they often appear in clusters or in close proximity.

The storage tank detection model achieved high performance, with slightly higher overall AP in the Permian basin and higher recall than precision in both basins (Table 4). The model accurately identifies the number of storage tanks per well pad, estimating within 0.082 storage tanks of the true count on average (MAE). On well pads with no ground truth storage tanks, the model rarely produces false positives (MAE 0.010), while on well pads with storage tanks the model is relatively more error-prone but still produces accurate counts (MAE 0.272). We note that despite their small size, storage tanks are less susceptible to the issues with small object detection mentioned previously, primarily due to their homogeneity and to the fact that they only appear on well pads within the context of this work, constraining the number of false positive detections.

**Table 4:** Test set results of the best storage tank detection model

	Average precision	Precision	Recall	Mean absolute error		
				All well pads	Well pads with no tanks	Well pads with tanks
Permian	0.989	0.965	0.972	0.072	0.009	0.220
Denver	0.981	0.957	0.963	0.101	0.011	0.360
Overall	0.986	0.962	0.968	0.082	0.010	0.272

Inspecting a sample of incorrect predictions, we found that the model sometimes failed to detect storage tanks whose color exhibits low contrast with the well pad color. False positives most commonly occurred when the model incorrectly identified other cylindrical equipment as storage tanks, such as vertical heater treaters, separators, and water disposal tanks.

As with the well pad model, we evaluated the generalization of the storage tank model to new regions, and observed that the model remained a reliable detector of storage tanks with slightly decreased performance in the Anadarko (>0.930 precision and recall), Uinta-Piceance (>0.900 precision and recall), and TX-LA-MS Salt (>0.850 precision and recall) basins, with substantially worse performance in the Appalachian basin (>0.500 precision and recall). Full results and dataset counts are shown in Supplementary Table 8.

To produce storage tank detections across the full Permian and Denver basins, we fed images of the verified well pads produced in the previous section to the model. The storage tank model produced a total of 175,996 detections, with the majority of detections observed in the Permian basin (83.6%). We estimate that 18.0% of well pads in the Permian basin and 23.2% of well pads in the Denver basin have storage tanks, and the mean number of storage tanks per well pad is slightly higher in the Permian (4.194) than in the Denver basin (3.397) (Supplementary Table 9).

We evaluated a sample of 10,000 detections (*n* = 5000 in each basin), and found that 96.9% and 95.9% of the detections were in fact storage tanks in the Permian and Denver basins respectively, whereas the remaining detections were false positives. Based on the sample, we estimate that the model identified >142,000 and 27,000 storage tanks in the Permian and Denver basins, respectively. We note that recall cannot be estimated at the basin-level because no comprehensive sources of storage tank data are available.

**Fig. 5:**
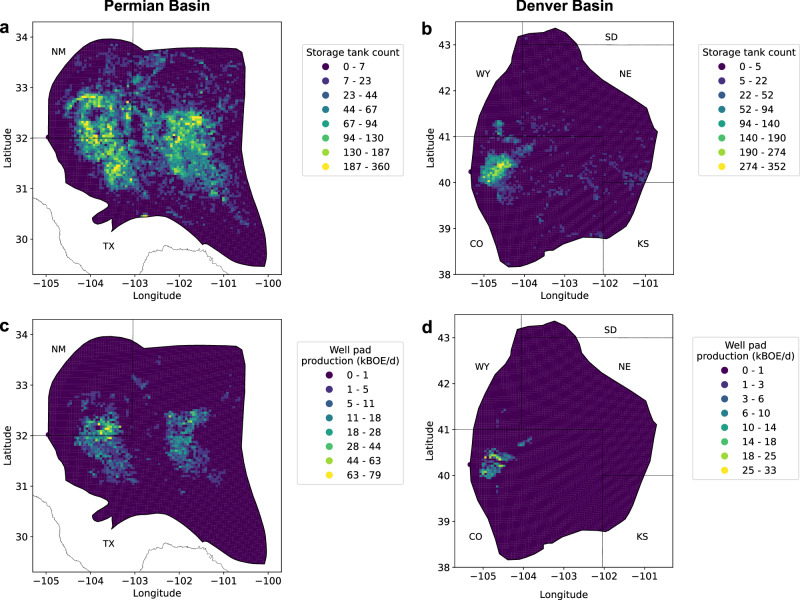
Basin-level gridded density heatmaps. Heatmaps show **a**, **b** storage tank counts and **c**, **d** well pad production measured in kilo barrels of oil equivalent per day (kBOE/d). Each grid cell represents 5 km^2^.

We also analyzed the relationship between storage tanks and well pad production of oil and natural gas, measured in kBOE/d. At the individual well pad level, we found a low correlation between storage tank count and kBOE/d (*r* = 0.20 in Permian, *r* = 0.22 in Denver). At a coarser level, however, storage tanks are largely most prevalent in production “hotspots” in both basins (Fig. 5). Additionally, when aggregating storage tank counts and production to 5 km^2^ cells, we found moderate correlation between counts and kBOE/d (*r* = 0.53 in Permian, *r* = 0.68 in Denver). We further stratified production into oil and gas production, measured in barrels of crude oil (BBL) and thousands of cubic feet (MCF) respectively, and found a higher correlation between tank counts and gas production (*r* = 0.55 in Permian, *r* = 0.72 in Denver) than oil production (*r* = 0.50 in Permian, *r* = 0.58 in Denver).

## Discussion

We show that modern machine learning methods on satellite imagery are effective for mapping O&G well pads and storage tanks in major U.S. basins. In particular, we demonstrate the methodology for training models on carefully curated labeled datasets of well pads and storage tanks in high-resolution imagery, and the application of those models at scale across the Permian and Denver basins.

We perform a number of experiments evaluating the performance of the models, demonstrating that a model trained jointly in the Permian and Denver basins achieves high precision (>0.95) and recall (>0.90) and outperforms basin-specific models. We found that the model performs somewhat worse in the Denver basin, which we attribute primarily to the prevalence of small well pads in the region, which are difficult to detect, and to a broader range of “built” urban and suburban infrastructure in the basin. We also demonstrate that a two-stage detection approach, wherein the first stage detects well pads while maximizing recall and the second stage verifies well pads while maximizing precision, improves the overall performance of the detection task. Further, we evaluate the model’s ability to generalize beyond the Permian and Denver basins, and show that the model maintains high performance in regions with low distribution shift, but suffers considerably in regions where well pads and the surrounding environment exhibit different visual characteristics than the training regions. These experiments suggest that context-specific training data is required to fine-tune the model in regions with higher distribution shifts. Finally, we train highly accurate (>0.96 precision and recall) storage tank detection models for both the Permian and Denver basins.

This study produced datasets of bounding boxes for all types of well pads (including both active/inactive and bare well pads where drilling may not have occurred). Additionally, this work produced a dataset of storage tanks on well pads, which to our knowledge is unavailable in both public and proprietary databases. The application of this work and the results obtained are significant as the Permian basin alone accounts for over 40% of US national oil production^28^. Based on the analysis of the well pad and storage tank data produced by our model deployment on high-resolution satellite imagery, we found distinct well-level activity hotspots in both the Permian and Denver basins that co-align with the basin-wide production hotspots and emission profiles in the two basins when compared with recently published independent data^29^. Such spatially explicit density heatmaps of O&G activity data can help better understand production and emission characteristics in major O&G basins globally.

Our well pad pipeline produces detections that match well with existing public and private datasets, while also producing >70,000 detections not found in those datasets, an increase of approximately 33%. Similarly, we detect >169,000 previously unidentified storage tanks across both basins. These new detections illustrate the potential of our methods for filling data gaps in order to perform more accurate emission estimates and source attribution. Further scaling this work to national and global scales could provide even more value, as many regions are not as well studied and documented as the basins in this work.

Our approach has some limitations. Most notably, we found a significant gap between the well pad model’s performance evaluated on our test set and during deployment. In the Permian, we achieved 0.975 precision and 0.906 recall on the test set and 0.944 precision and 0.805 recall during deployment; in the Denver, we achieved 0.935 precision and 0.901 recall on the test set and 0.720 precision and 0.681 recall during deployment. The high performance on the test set and shift in performance between the test and deployment settings are comparable to other work using deep learning to map energy infrastructure^25,38^; below we describe some reasons for this shift and strategies for reducing it.

First, we note that our manually-curated test set contained high-quality well pad annotations, whereas the Enverus and HIFLD datasets used for evaluation during deployment are noisy and may contain inaccurate or outdated locations (Supplementary Fig. 4). Therefore, the deployment metrics (in particular, recall) underestimate true model performance.

Beyond the quality of the reported datasets, we attribute this gap to the distribution shift between the two settings. There exists some label bias in our training dataset, as 50% of the labels were collected by domain experts who manually panned around the basins and generally annotated “prototypical” well pads (e.g. larger well pads with clearly defined footprints). The other 50% of labels were created by randomly sampling wells completed post-2005 (chosen to maximize the number of actual well pads contained in the sample). While the latter strategy reduces bias, it does not eliminate it entirely; well pads older than 2005 are not well-represented in the training dataset despite comprising a large portion of the actual distribution of well pads in both basins.

Further, both sampling strategies were biased towards selecting larger well pads (well pad size grew rapidly post-2005, as shown in Fig. 3). This may in part explain the high correlation between the performance of the well pad detection pipeline and well pad size, as the model struggles to detect smaller, single wellhead pads. This issue is particularly clear in the Denver basin, where small well pads are more prevalent and the precision and recall of the model are significantly lower than in the Permian basin on the evaluation sets and during deployment.

These shortcomings could be addressed by randomly sampling the entire set of labels across all years to better reflect the true distribution of well pads in the training dataset, including more accurately representing older/smaller well pads, which could help close the gap in performance between evaluation and deployment. Additionally, the diversity of positives and negatives seen during a large-scale deployment at the basin level is likely higher than what was captured in our training dataset, leading to false positives and negatives. Solutions include collecting a larger dataset, and iterative deployments where false positives and negatives are incorporated into the training dataset after each iteration before retraining the model.

Outdated imagery also limited our ability to detect recently constructed well pads, which are responsible for the large majority of overall production. We found relatively high agreement between our detections and reported well pad datasets on well pads constructed from 2010–2016, and low agreement from 2017–2021, which we attribute primarily to outdated imagery (acquired before the well pad construction date) in the Google Earth satellite basemap. While other publicly available sources of imagery exist with more recent coverage, they lack in spatial resolution (e.g., Landsat 8^39^, 15 m) or geographic scope (e.g. NAIP^40^ only captures the contiguous US) for mapping recently constructed well pads reliably and scalably. Despite this limitation, our ability to fill gaps in the mapping of older, lower-producing well pads is important, as previous work showed that such well pads account for a disproportionately large amount of methane emissions^41^. Future work should explore leveraging other commercial imagery sources that have high spatial resolution, revisit rate, and global coverage like Airbus SPOT^42^ (1.5 m) or PlanetScope^43^ (3 m).

Addressing the challenges mentioned above would enable a globally scalable well pad and storage tank mapping framework, which could prove especially useful in high-producing countries with little transparent O&G infrastructure data (e.g. Russia). Other future directions include applying our framework to detect other well pad features such as pump jacks and flares, and other methane-emitting facilities such as natural gas compressor stations and oil terminals. These directions would further contribute to constructing a large-scale, granular, and accurate geospatial database of O&G infrastructure, a key ingredient in measuring and mitigating methane emissions across the global O&G sector.

## Methods

### Training dataset for well pads

We framed well pad detection as an object detection task, wherein models are trained to input an image and output axis-aligned bounding boxes around instances of well pads. To train and validate the models, we first collected a labeled dataset of images containing known well pads in the Permian and Denver basins. Although public and private O&G well databases exist, they are insufficient as a source of training labels. These databases are limited to point locations of wells, while bounding boxes around well pads are necessary for training deep-learning object detection models to output such bounding boxes. Furthermore, not every image capturing a well from an O&G database will contain an actual well pad for a variety of reasons: (1) old wells may be decommissioned by the image capture date and no longer visible in the image, (2) new wells may not be constructed by the image capture date and not yet present in the image, or (3) the well locations may be imprecise. To create bounding box labels for the models which capture well pads, we obtained labels via (a) domain experts and (b) crowd-sourcing, as further described below.

For (a), co-authors with expertize identifying O&G facilities and associated equipment manually panned around the basins using the Google Earth^33^ satellite basemap (30-70 cm spatial resolution in the US) in QGIS^44^, an open-source geographic information system (GIS) application. The experts annotated bounding boxes around well pads and storage tanks, then saved the coordinates using a QGIS plugin. We note that these labels are high-quality, but were selected via manual panning of the basins, and thus cannot be considered a representative sample of well pads in the regions. We address this with (b) below.

For (b), we uploaded images centered at potential well pad locations to Scale AI^45^, a crowd-sourced data labeling service. We obtained these locations from public (HIFLD^16^) and private (Enverus^35^ 2021) O&G well databases. We filtered the well databases for active wells with construction completion dates post-2005 and randomly sampled 5,900 locations from the databases. We chose these filters to increase the probability that a sampled location would actually contain a well pad, thereby maximizing the number of labels we could produce (inactive and/or older wells in the databases more often have inaccurate coordinates or no well pad visible in satellite imagery). Images of these locations were then uploaded to Scale AI for labeling; 81% of the images contained well pads. As non-experts labeled this data, the annotations are lower-quality, but because they were randomly sampled from well data they are more representative of the full distribution of well pads and surrounding landscapes. To ensure high quality of the datasets used for evaluation, we manually reviewed the validation and test sets (described below) to remove false positives and occasionally correct poorly drawn boxes.

The full well pad training dataset consisted of 10,432 images containing 12,490 well pads (some images contained multiple well pads). Approximately 50% of labels came from each of the data labeling procedures described above.

We sampled negative examples from a variety of sources in order to train a highly discriminative model. We first sampled random locations within each basin, as well as random locations within city boundaries in each basin. These negatives mostly bear low visual similarity to well pads, and are insufficient for training a discriminative model. In order to collect more “difficult” negatives with increased visual similarity to well pads, we launched a preliminary deployment of an early well pad detection model in the basins and manually identified common objects and landscapes that were incorrectly classified by the model as well pads including roads, wind turbines, lake beds, river banks, exposed soil, and agricultural fields. We sampled locations of roads and wind turbines from the OpenStreetMap database and included them in the final set of negative locations. For the other landscapes which led to false positives, we used an open-source GeoVisual similarity search tool to collect a large amount of images containing visually similar landscapes^46^. Several of these images were collected from outside the Permian and Denver basins, which is reflected in Supplementary Table 10.

We downloaded 77,612 negative examples from these various sources, for an approximately 1:7 positive to negative ratio. We constructed 640 × 640 pixel tiles and projected the images to Web Mercator at zoom level 1600, which corresponds to ~197 m × 197 m in the Denver basin, and ~223 m × 223 m in the Permian basin.

Finally, we randomly split the dataset into a training set (75%) to identify the model parameters, a validation set (15%) to tune model hyperparameters, and a testing set (10%) to evaluate the best model. We also ensured that any overlapping images appear in the same split to prevent data leakage. Full dataset counts by split and basin are shown in Supplementary Table 10 and dataset samples of positive and negatives in Supplementary Fig. 5.

To evaluate model performance in new regions that were not seen during training, we collected additional well pad datasets in the Appalachian, TX-LA-MS Salt, Anadarko, and Uinta-Piceance basins. These datasets were curated in a similar manner to the Permian and Denver dataset, though positives were labeled solely through crowdsourcing and were manually reviewed. Full dataset counts and dataset samples of positives are shown in Supplementary Table 5 and Supplementary Fig. 3.

### Model training and evaluation

Neural networks are a class of models with parameters organized into several layers, where each layer of the model extracts relevant features from the input at varying levels of abstraction, and uses these features to produce an output prediction. We trained a convolutional neural network (CNN) -- a particular type of neural network designed for image data – to input a satellite image and output a list of bounding boxes representing predicted well pad locations (if any) in the image, a well-established computer vision task called object detection.

We trained the model using a standard optimization procedure as follows: we input batches of satellite images into the model and compute a loss value that measures how well the output predictions match the annotated well pads described in the previous section. The model parameters are then updated using stochastic gradient descent (SGD), where the update for each parameter consists of the gradient with respect to the loss multiplied by a small “step size” in the direction that minimizes the loss. This procedure was repeated iteratively until the loss converged, thereby training the model to more closely match the annotated well pads. The model architecture, loss function, number of images processed per iteration (referred to as “batch size”), and step size (referred to as “learning rate”) are tunable hyperparameters. We describe the various settings we tried below.

We experimented with various object detection architectures (Faster RCNN^36^, RetinaNet^30^, SSD^47^, YOLOv3^48^) and backbones (ResNet^31^, RegNet^49^, ResNeSt^50^, Res2Net^37^, EfficientNet^32^ variations) and determined the highest-performing model to be the single-stage RetinaNet detector with a ResNet-50 backbone. The model was initialized with pretrained ImageNet^51^ weights, and was trained end-to-end using Absolute Error Loss and Focal Loss^30^ for bounding box regression and classification, respectively.

We trained our model on 512 × 512 images using the Adam optimizer^52^ (a modified form of SGD) with learning rate 1e-6 and batch size 8 on a single NVIDIA RTX A4000 GPU. During training, we performed stochastic image augmentations, including random crops, flips, scaling, and lighting/saturation jitter to increase the model’s robustness to natural variation in the satellite imagery. We performed random cropping and scaling during evaluation in order to simulate the distribution of well pads during deployment, where well pads are rarely at the center of the image. Because the imbalance between the number of positive and negative examples in the dataset can lead to poor model behavior, we sampled positives and negatives with weights inversely proportional to their frequencies in the dataset. We also experimented with training basin-specific detection models in the Permian and Denver basins versus training a model jointly in both basins.

Additionally, we removed all ground truth labels and predictions whose centerpoint was within 50 pixels of the image border, as these are generally well pads that are only slightly visible within the image. We evaluated the model on the validation set after each epoch, and saved the checkpoint with the highest AP.

We evaluated the detection models using a variety of standard object detection metrics. For each image, the detection models output a list of bounding boxes in the image after non-maximum suppression, which eliminates predicted boxes with high overlap and retains only the highest confidence box. Each box is associated with coordinates and a confidence score between 0 and 1 indicating the likelihood that the box contains a well pad.

We evaluated the models at fixed confidence thresholds using precision (the proportion of model predictions which are actually well pads) and recall (the proportion of actual well pads in the dataset that were correctly identified by the model). After fixing a confidence threshold, any predicted boxes assigned a score higher than the threshold are kept and boxes with a score lower than the threshold are discarded. To determine whether a model’s predicted bounding box matched the ground truth bounding box, we use an Intersection over Union (IoU) threshold of 0.3 and greedily match boxes until exhausting the predicted boxes. Matched boxes are true positives, unmatched predicted boxes are false positives, and unmatched ground truth boxes are false negatives. Precision and recall are then computed as described above. We note that the IoU threshold of 0.3 used here is lower than typical threshold values typically used for evaluation in object detection (0.5–0.95). We justify the choice of this threshold based on ambiguity in consistently defining well pad boundaries (Supplementary Fig. 6).

To evaluate the performance of the model across all confidence thresholds, we vary the threshold between 0 and 1, and compute the precision and recall at all thresholds to construct a precision-recall curve. The area under this curve, also known as average precision (AP), was used as a single metric to summarize the detection performance across all thresholds, where the lowest value of the metric is 0 and the highest is 1.

In addition to AP, we specifically measured performance on the validation set at thresholds corresponding to 95% recall in the Permian basin and 93% recall in the Denver basin in order to increase the completeness of the dataset when the model is deployed. We show the selected threshold values and the precision-recall curve of the detection model in Supplementary Fig. 7.

Because we used stochastic augmentations during validation, we determined this threshold by calculating the mean over 10 runs. We report the mean and standard deviation of other relevant metrics across these 10 runs.

### Well pad verification

In addition to the well pad detection model, we trained and validated a well pad verification model, whose purpose is to serve as a dedicated model for verifying individual instances of well pads detected by the detection model. The hypothesis was that a model specialized for an individual well pad verification task would help reduce false positive detections produced by the detection model, which is often tasked to predict multiple well pads in a single image. The model performs binary classification to determine whether or not a satellite image contains a well pad. During deployment, well pad detections that were verified by this model were considered “fully verified” and retained in the final dataset, while detections that were not verified were discarded.

The model was trained on a similar well pad dataset as described for well pad detection, but for every well pad annotation we downloaded an image centered at the centerpoint of the bounding box. We used the same set of negatives as for well pad detection.

As with the well pad detection model, we experimented with several model backbones (ResNet, EfficientNet, ResNeXt^53^, Inception^54^, DenseNet^55^ variants) and hyperparameters, and found EfficientNet-B3 to be the highest performing architecture. We also tuned hyperparameters for learning rate, oversampling of positive examples, and data augmentations. The model was trained with a 1e-4 learning rate, and the same optimization, GPU, oversampling, and training data augmentation settings described in the previous section, with no stochastic augmentations during evaluation because well pads were guaranteed to be centered at this stage.

The model was evaluated against the validation set and checkpointed using F1 score. Because well pad detections verified by this model were considered “fully verified,” we chose a threshold corresponding to 99% precision on the validation set to minimize false positives in the final dataset. Because no stochastic augmentations were used during evaluation, the metrics were deterministic at this stage.

To evaluate the detection model in conjunction with the verification model, we downloaded images centered at every candidate prediction to feed through the verification model, and candidates that were not “verified” were removed from the predictions. We used AP, precision and recall to evaluate the remaining “verified” detection predictions.

### Storage tank detection

We also trained and validated a storage tank detection model, framed as an object detection task, where the model outputs an axis-aligned box for each storage tank on a well pad. We obtained labeled storage tanks through the two annotation procedures described for the well pad detection dataset. We note that while the number of images with storage tanks in the dataset is relatively small for a deep learning task (1,833), each well pad can contain several storage tanks, so the total number of storage tanks is 10,470. Negative examples are simply well pads without storage tanks. We did not require the same diversity of negatives as in the well pad detection and verification tasks because the storage tank model is only deployed on detected well pads. Full dataset counts by split and basin are shown in Supplementary Table 11 and dataset samples with storage tank annotations in Supplementary Fig. 8. To evaluate the generalization of the model, we collected storage tank datasets in the same evaluation basins previously described for well pad detection by labeling the storage tanks on well pads in the basins.

We experimented with the same architectures and backbones described in the well pad detection section, and found the two-stage FasterRCNN^36^ architecture with a Res2Net^37^ backbone to be the highest-performing model. We also tuned hyperparameters for learning rate and anchor box scale due to the small size of storage tanks. The model was trained with a 5e-5 learning rate, [4,6,8] anchor box scales, and the same optimization, GPU, and data augmentation settings described in the well pad detection section, with no augmentations during evaluation because well pads were guaranteed to be centered at this stage.

We evaluated the models using the same metrics described in the well pad detection section. In addition, we measured the mean absolute error (MAE) between ground truth and predicted storage tank counts across well pads containing ground truth storage tanks, well pads containing no ground truth storage tanks, and across all well pads. We thresholded the model at values that maximized the F1 score in both basins; we show the selected threshold values and the precision-recall curve of the model in Supplementary Fig. 7. Because no stochastic augmentations were used during evaluation, the metrics were deterministic at this stage.

### Deployment

**Fig. 6:**
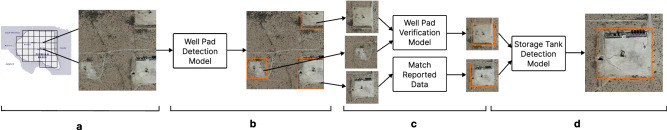
Overall tiling, well pad detection, verification, and storage tank detection process. **a** The deployment region was tiled into smaller images, **b** each of which were input to the well pad detection model which produced bounding boxes indicating the location of potential well pads. **c** Images around the candidate well pads were verified if they matched reported data or if they were confirmed by the verification model. **d** Finally, images around the verified well pads were input to the storage tank detection model to identify the locations of any storage tanks on the pads.

In this section, we detail the procedure for deploying trained well pad detection and storage tank models in the Permian and Denver basins (a visual explanation is shown in Fig. 6). Briefly, we tiled each basin into a collection of images, which were fed through the well pad detection model. Detections were verified either through the well pad verification model or by matching reported data. Finally, images with verified well pads were fed through the storage tank detection model to obtain finalized well pad and storage tank datasets.

We tiled both basins into 512 × 512 images, and projected the images to Web Mercator at zoom level 1600 (~197 × 197 m in the Denver basin ~223 × 223 m in the Permian basin). We also overlapped the tiles by 100 pixels in order to minimize instances where well pads were at the edge of images, which are difficult to detect. We tiled and downloaded 7.1 million images in the Permian basin and 6.8 million images in the Denver basin through this procedure, spanning a total area of 313,340 km^2^ across both basins.

We fed each image tile through the well pad detection model and applied non-maximum suppression with an IoU of 0.2 to produce a set of candidate detections. Inference took ~14 h to complete on 4 NVIDIA RTX A4000 GPUs, with a total batch size of 96. We then performed post processing to convert candidate detections from pixel-based to latitude/longitude coordinates, merge overlapping detections, and drop candidate detections whose confidence score was less than the recall thresholds calculated during model validation.

Once the candidate well pad detections were post-processed, we downloaded a new image for every candidate detection centered at the well pad, and fed these images through the well pad verification model. For well pads larger than the default image size, we downloaded images at a higher zoom level. Candidates whose confidence scores were higher than the 99% precision threshold were considered to be verified by the verification model.

We also verified candidate detections by matching them to reported data in the HIFLD and Enverus well datasets. We considered other commonly known O&G infrastructure data repositories such as OGIM^19^ (v1.1) and GOGI^56^ (v10.3.1) but we did not use them in this study as the former sources exclusively from HIFLD in the Permian and Denver basins, and the latter primarily consists of gridded well counts rather than point locations.

We spatially clustered the wells in each dataset to form well pad datasets (described in detail in Supplementary Note 1). We then buffered candidate detections by 50 meters to account for any inaccuracies in the point locations, and performed a spatial join between the candidate detections and reported well pads. Candidates that contain a reported well pad point location were considered to be verified by the reported data. Finally, we dropped candidates that were not verified by either the verification model or reported data. The remaining well pads were considered the fully verified dataset.

Lastly, we fed images of the fully verified well pads to the storage detection model and post-processed the detections in the same manner described for well pads. Because the storage detection model was highly accurate and no sources of reported data exist, we did not perform verification on the storage tank detections.

### Supplementary information

Supplementary Information

Peer Review File

### Source data

Source Data

## Data Availability

The datasets curated to train the well pad and storage tank models and those generated through the basin-scale deployments have been deposited at: 10.5281/zenodo.11660152. We are unable to redistribute the satellite imagery used to train the models in this study due to data licensing, but these data may be freely accessed directly from the original provider. Source data are provided with this paper.

## Code availability

The code written to evaluate the models is publicly available at: https://github.com/stanfordmlgroup/well-pad-denver-permian. Code for training the models may be made available upon request to the corresponding author.

## References

[1] Methane and climate change – Global Methane Tracker 2022 – Analysis. *IEA* https://www.iea.org/reports/global-methane-tracker-2022/methane-and-climate-change (2022).

[2] Chapter 7: The Earth’s Energy Budget, Climate Feedbacks, and Climate Sensitivity. https://www.ipcc.ch/report/ar6/wg1/chapter/chapter-7/ (2021).

[3] US Department of Commerce, N. Global Monitoring Laboratory - Carbon Cycle Greenhouse Gases. https://gml.noaa.gov/ccgg/trends_ch4/ (2024).

[4] Jackson, R. B. et al. Increasing anthropogenic methane emissions arise equally from agricultural and fossil fuel sources. *Environ. Res. Lett.* **15**, 071002 (2020). 10.1088/1748-9326/ab9ed2

[5] Environment, U. N. Global Methane Assessment: Benefits and Costs of Mitigating Methane Emissions. *UNEP - UN Environment Programme* http://www.unep.org/resources/report/global-methane-assessment-benefits-and-costs-mitigating-methane-emissions (2021).

[6] Nisbet, E. G. et al. Methane mitigation: methods to reduce emissions, on the path to the paris agreement. *Rev. Geophys.* **58**, e2019RG000675 (2020). 10.1029/2019RG000675

[7] Nisbet, E. G. et al. Very strong atmospheric methane growth in the 4 years 2014–2017: implications for the Paris agreement. *Glob. Biogeochem. Cycles* **33**, 318–342 (2019). 10.1029/2018GB006009

[8] Ocko, I. B. et al. Acting rapidly to deploy readily available methane mitigation measures by sector can immediately slow global warming. *Environ. Res. Lett.* **16**, 054042 (2021). 10.1088/1748-9326/abf9c8

[9] Omara, M. et al. Methane emissions from natural gas production sites in the united states: data synthesis and national estimate. *Environ. Sci. Technol.* **52**, 12915–12925 (2018). 30256618 10.1021/acs.est.8b03535

[10] Lyon, D. R. et al. Aerial surveys of elevated hydrocarbon emissions from oil and gas production sites. *Environ. Sci. Technol.* **50**, 4877–4886 (2016). 27045743 10.1021/acs.est.6b00705

[11] Reconciling divergent estimates of oil and gas methane emissions | PNAS. https://www.pnas.org/doi/abs/10.1073/pnas.1522126112 (2015). 10.1073/pnas.1522126112 PMC4697433 26644584

[12] ACPD - Quantification of Oil and Gas Methane Emissions in the Delaware and Marcellus Basins Using a Network of Continuous Tower-Based Measurements. https://acp.copernicus.org/preprints/acp-2022-709/ (2023).

[13] Jacob, D. J. et al. Quantifying methane emissions from the global scale down to point sources using satellite observations of atmospheric methane. *Atmos. Chem. Phys.* **22**, 9617–9646 (2022). 10.5194/acp-22-9617-2022

[14] Jacob, D. J. et al. Satellite observations of atmospheric methane and their value for quantifying methane emissions. *Atmos. Chem. Phys.* **16**, 14371–14396 (2016). 10.5194/acp-16-14371-2016

[15] Rafiq, T. et al. Attribution of methane point source emissions using airborne imaging spectroscopy and the Vista-California methane infrastructure dataset. *Environ. Res. Lett.* **15**, 124001 (2020). 10.1088/1748-9326/ab9af8

[16] HIFLD Open Data. https://hifld-geoplatform.opendata.arcgis.com/ (2019).

[17] National Academies of Sciences, E. et al. *Current Inventories of Methane Emissions*. *Improving Characterization of Anthropogenic Methane Emissions in the United States* (National Academies Press (US), 2018). 30110140

[18] Rutherford, J. S. et al. Closing the methane gap in US oil and natural gas production emissions inventories. *Nat. Commun.* **12**, 4715 (2021). 34354066 10.1038/s41467-021-25017-4 PMC8342509

[19] Omara, M. et al. Developing a spatially explicit global oil and gas infrastructure database for characterizing methane emission sources at high resolution. *Earth Syst. Sci. Data Discuss*. 10.5194/essd-2022-452 (2023).

[20] Li, W. et al. Semantic segmentation-based building footprint extraction using very high-resolution satellite images and multi-source GIS data. *Remote Sens.* **11**, 403 (2019). 10.3390/rs11040403

[21] Zhang, P. et al. Urban land use and land cover classification using novel deep learning models based on high spatial resolution satellite imagery. *Sensors* **18**, 3717 (2018). 30388781 10.3390/s18113717 PMC6263528

[22] Ren, S. et al. Automated extraction of energy systems information from remotely sensed data: a review and analysis. *Appl. Energy* **326**, 119876 (2022). 10.1016/j.apenergy.2022.119876

[23] Yu, J., Wang, Z., Majumdar, A. & Rajagopal, R. DeepSolar: a machine learning framework to efficiently construct a solar deployment database in the United States. *Joule* **2**, 2605–2617 (2018). 10.1016/j.joule.2018.11.021

[24] Zhou, S. et al. DeepWind: Weakly Supervised Localization of Wind Turbines in Satellite Imagery. In *NeurIPS 2019 Workshop on Tackling Climate Change with Machine Learning* (2019).

[25] Sheng, H. et al. OGNet: towards a global oil and gas infrastructure database using deep learning on remotely sensed imagery. In *NeurIPS 2020 Workshop on Tackling Climate Change with Machine Learning* (2020).

[26] Zhu, B. et al. METER-ML: a multi-sensor earth observation benchmark for automated methane source mapping. Preprint at http://arxiv.org/abs/2207.11166 (2022).

[27] Dileep, S., Zimmerle, D., Beveridge, R. & Vaughn, T. *Climate Change AI* (Climate Change AI, 2020).

[28] Advances in technology led to record new well productivity in the Permian Basin in 2021. https://www.eia.gov/todayinenergy/detail.php?id=54079 (2022).

[29] Zhang, Y. et al. Quantifying methane emissions from the largest oil-producing basin in the United States from space. *Sci. Adv.* **6**, eaaz5120 (2020). 32494644 10.1126/sciadv.aaz5120 PMC7176423

[30] Lin, T.-Y., Goyal, P., Girshick, R., He, K. & Dollár, P. Focal loss for dense object detection. In *Proceedings of the IEEE international conference on computer vision* 2980–2988 (2017).

[31] He, K., Zhang, X., Ren, S. & Sun, J. Deep Residual Learning for Image Recognition. In *2016 IEEE Conference on Computer Vision and Pattern Recognition (CVPR)* 770–778 (IEEE, 2016).

[32] Tan, M. & Le, Q. EfficientNet: rethinking model scaling for convolutional neural networks. In *Proceedings of the 36th International Conference on Machine Learning* 6105–6114 (PMLR, 2019).

[33] Google Earth. https://earth.google.com/web/ (2024).

[34] Liu, Y., Sun, P., Wergeles, N. & Shang, Y. A survey and performance evaluation of deep learning methods for small object detection. *Expert Syst. Appl.* **172**, 114602 (2021). 10.1016/j.eswa.2021.114602

[35] Enverus. https://www.enverus.com/ (2021).

[36] Ren, S., He, K., Girshick, R. & Sun, J. Faster R-CNN: towards real-time object detection with region proposal networks. In *Advances in Neural Information Processing Systems* vol. 28 (Curran Associates, Inc., 2015).

[37] Gao, S.-H. et al. Res2Net: a new multi-scale backbone architecture. *IEEE Trans. Pattern Anal. Mach. Intell.* **43**, 652–662 (2021). 31484108 10.1109/TPAMI.2019.2938758

[38] Kruitwagen, L. et al. A global inventory of photovoltaic solar energy generating units. *Nature* **598**, 604–610 (2021). 34707304 10.1038/s41586-021-03957-7

[39] Landsat 8 | Landsat Science. https://landsat.gsfc.nasa.gov/satellites/landsat-8/ (2021).

[40] National Agriculture Imagery Program - NAIP Hub Site. https://naip-usdaonline.hub.arcgis.com/ (2024).

[41] Omara, M. et al. Methane emissions from US low production oil and natural gas well sites. *Nat. Commun.* **13**, 2085 (2022). 35440563 10.1038/s41467-022-29709-3 PMC9019036

[42] Satellite Imagery | Earth Observation | Airbus Space. https://www.airbus.com/en/space/earth-observation/satellite-imagery (2021).

[43] Planet | Homepage. *Planet* https://www.planet.com/ (2024).

[44] QGIS. https://qgis.org/en/site/ (2024).

[45] Scale AI. https://scale.com/ (2024).

[46] Keisler, R. et al. Visual search over billions of aerial and satellite images. *Comput. Vis. Image Underst.* **187**, 102790 (2019). 10.1016/j.cviu.2019.07.010

[47] Liu, W. et al. *SSD: Single Shot Multibox Detector*. Vol. 9905.p. 21–37 (ECCV, 2016).

[48] Redmon, J. & Farhadi, A. YOLOv3: An incremental improvement. Preprint at 10.48550/arXiv.1804.02767 (2018).

[49] Radosavovic, I., Kosaraju, R. P., Girshick, R., He, K. & Dollar, P. Designing network design spaces. In *2020**IEEE/CVF Conference on Computer Vision and Pattern Recognition (CVPR)*. p. 10425–10433 (IEEE, 2020).

[50] Zhang, H. et al. ResNeSt: split-attention networks. in *2022**IEEE/CVF Conference on Computer Vision and Pattern Recognition Workshops (CVPRW).* p. 2735–2745 (IEEE, 2022).

[51] Deng, J. et al. ImageNet: A large-scale hierarchical image database. in *2009 IEEE Conference on Computer Vision and Pattern Recognition.* p. 248–255 (2009).

[52] Kingma, D. P. & Ba, J. Adam: a method for stochastic optimization. Preprint at 10.48550/arXiv.1412.6980 (2017).

[53] Xie, S., Girshick, R., Dollar, P., Tu, Z. & He, K. Aggregated residual transformations for deep neural networks. In *2017 IEEE Conference on Computer Vision and Pattern Recognition (CVPR).* p. 5987–5995 (IEEE, 2017).

[54] Szegedy, C. et al. Going deeper with convolutions. In *2015 IEEE Conference on Computer Vision and Pattern Recognition (CVPR)* 1–9 (IEEE, 2015).

[55] Huang, G., Liu, Z., Van Der Maaten, L. & Weinberger, K. Q. Densely connected convolutional networks. In *2017 IEEE Conference on Computer Vision and Pattern Recognition (CVPR)* 2261–2269 (IEEE, 2017).

[56] Global Oil & Gas Features Database - EDX. https://edx.netl.doe.gov/dataset/global-oil-gas-features-database (2017).

