# Supplementary material for: Deep learning for detecting and characterizing oil and gas well pads in satellite imagery
Source: Nat Commun. 2024 Aug 15;15:7036. doi: 10.1038/s41467-024-50334-9 (PMC11327246; doi:10.1038/s41467-024-50334-9)
Supplement: Supplementary file 1 — Supplementary Information [file 41467_2024_50334_MOESM1_ESM.pdf]

Supplementary Information for

Deep learning for detecting and characterizing oil and gas well pads in satellite imagery

Neel Ramachandran, Jeremy Irvin, Mark Omara, Ritesh Gautam, Kelsey Meisenhelder, Erfan Rostami, Hao Sheng, Andrew Y. Ng, Robert B. Jackson

This PDF file includes:

Supplementary Table 1 to 12
Supplementary Fig. 1 to 8
Supplementary Note 1

Supplementary Table 1. Validation set results across several architectures and backbones for the well pad detection model. The highest-performing model, shown in bold, was selected for the basin-scale deployments. The results are reported as the mean and standard deviation (SD) across 10 runs.

Architecture	Backbone	Average Precision (Mean ± SD)
SSD	VGG-16	0.844 ± 0.003
YoloV3	DarkNet-53	0.834 ± 0.004
RetinaNet	ResNet-50	**0.980 ± 0.001**
	ResNet-101	0.971 ± 0.002
	ResNeXt-101	0.972 ± 0.002
	RegNetX-3.2GF	0.973 ± 0.002
	ResNest-50	0.953 ± 0.002
	Res2Net-101	0.923 ± 0.002
	EfficientNet-b3	0.972 ± 0.002
FasterRCNN	ResNet-50	0.974 ± 0.003
	ResNet-101	0.970 ± 0.002
	ResNeXt-101	0.970 ± 0.003
	RegNetX-3.2GF	0.972 ± 0.002
	ResNest-50	0.974 ± 0.002
	Res2Net-101	0.977 ± 0.001

Supplementary Table 2. Validation set results across several backbones for the well pad verification model. The highest-performing model, shown in bold, was selected for the basin-scale deployments.

Backbone	Overall F1
Inception-v3	0.993
ResNet-50	0.996
ResNet-101	0.996
ResNeXt-101	0.997
DenseNet-121	0.998
EfficientNet-b3	**0.999**

Supplementary Table 3: Validation set results in the Permian and Denver basins with the standalone detection model, and with the detection and verification models (i.e. the full detection pipeline). We note that the Average Precision of the former cannot be fairly compared to the latter: Any falsely unverified true positives are filtered out by the verification model, introducing an upper bound on achievable recall which penalizes Average Precision at high thresholds. Thus, we only use precision and recall at a single threshold to evaluate the verified detection predictions. The results are reported as the mean and standard deviation (SD) across 10 runs.

		Precision (Mean ± SD)	Recall (Mean ± SD)
Permian	Detection Model	0.962 ± 0.004	0.932 ± 0.001
	Detection and Verification Models	**0.975 ± 0.002**	0.932 ± 0.003
Denver	Detection Model	0.937 ± 0.007	0.895 ± 0.001
	Detection and Verification Models	**0.946 ± 0.004**	0.895 ± 0.005

Supplementary Table 4. **Validation set results (Average Precision) of the well pad detection model.** Comparison of models trained in individual basins compared to the model jointly trained in both basins (bold), which achieves the highest performance. The results are reported as the mean and standard deviation (SD) across 10 runs.

	Validation Set	
Training Set	Permian (Mean ± SD)	Denver (Mean ± SD)
Permian	0.986 ± 0.001	0.872 ± 0.007
Denver	0.861 ± 0.003	0.963 ± 0.004
Both	**0.990 ± 0.001**	**0.969 ± 0.003**

Supplementary Table 5: Well pad detection model performance and dataset counts in the evaluation basins. Results from the Permian/Denver test set (from Table 1) are shown for comparison. We note that the results shown in the evaluation basins were produced solely by the detection model, as the verification model did not improve model performance, while the results shown in the Permian/Denver regions leverage the verification model. As such, Average Precision cannot fairly be compared between the Permian/Denver regions and the evaluation basins and is not presented here (see Supplementary Table 3**)**. The results are reported as the mean and standard deviation (SD) across 10 runs.

Basin	Precision (Mean ± SD)	Recall (Mean ± SD)	# Positive Images	# Negative Images
Permian/Denver	0.955 ± 0.004	0.904 ± 0.006	1,202	7,764
Appalachian	0.647 ± 0.031	0.552 ± 0.020	574	4,111
TX-LA-MS Salt	0.866 ± 0.016	0.806 ± 0.014	614	4,045
Anadarko	0.906 ± 0.016	0.856 ± 0.019	676	4,121
Uinta-Piceance	0.948 ± 0.009	0.943 ± 0.010	625	4,329

Supplementary Table 6. Percent of total area covered by acquisition year for the Google Earth satellite basemap in the Permian and Denver basins.

	% Total Area Covered	
Year	Permian	Denver
2013	0	19.30
2014	0.04	0.31
2015	0.34	16.55
2016	9.30	32.40
2017	21.65	0.43
2018	21.00	0.50
2019	47.66	30.50

Supplementary Table 7. Validation set results across several architectures and backbones for the storage tank detection model. The highest-performing model, shown in bold, was selected for the basin-scale deployments.

Architecture	Backbone	Average Precision
SSD	VGG-16	0.969
YoloV3	DarkNet-53	0.973
RetinaNet	ResNet-50	0.955
	ResNet-101	0.599
	ResNeXt-101	0.957
	RegNetX-3.2GF	0.952
	ResNest-50	0.312
	Res2Net-101	0.954
	EfficientNet-b3	0.952
FasterRCNN	ResNet-50	0.965
	ResNet-101	0.960
	ResNeXt-101	0.968
	RegNetX-3.2GF	0.966
	ResNest-50	0.973
	Res2Net-101	**0.977**

Supplementary Table 8: Storage tank detection model performance and dataset counts in the evaluation basins.

Basin	Average Precision	Precision	Recall	# Positive Images	# Negative Images
Permian/Denver	0.986	0.962	0.968	162	380
Appalachian	0.505	0.517	0.550	405	222
TX-LA-MS Salt	0.876	0.880	0.851	399	229
Anadarko	0.960	0.935	0.939	520	72
Uinta-Piceance	0.943	0.904	0.906	461	83

Supplementary Table 9. Deployment results of the best storage tank detection model. The mean number of storage tanks per well pad was calculated across well pads containing storage tanks.

	Total # of Detected Storage Tanks	Total # of Well Pads with Detected Storage Tanks	Total # of Well Pads without Detected Storage Tanks	Mean # of Storage Tanks per Well Pad
Permian	147,116	35,077	159,896	4.194
Denver	28,880	8,502	28,089	3.397
Overall	175,996	43,579	187,985	4.039

Supplementary Table 10. **Dataset counts for the well pad detection task in the Permian and Denver basins.**

	Training		Validation		Testing	
	# Positive Images	# Negative Images	# Positive Images	# Negative Images	# Positive Images	# Negative Images
Permian	4,499	17,512	1,106	3,481	739	2,267
Denver	2,987	17,275	638	3,545	463	2,433
Other	N/A	23,436	N/A	4,599	N/A	3,064

Supplementary Table 11. **Dataset counts for the storage tank detection task in the Permian and Denver basins.**

	Training		Validation		Testing	
	# Positive Images	# Negative Images	# Positive Images	# Negative Images	# Positive Images	# Negative Images
Permian	786	1,581	130	332	92	213
Denver	632	1,080	123	229	70	167

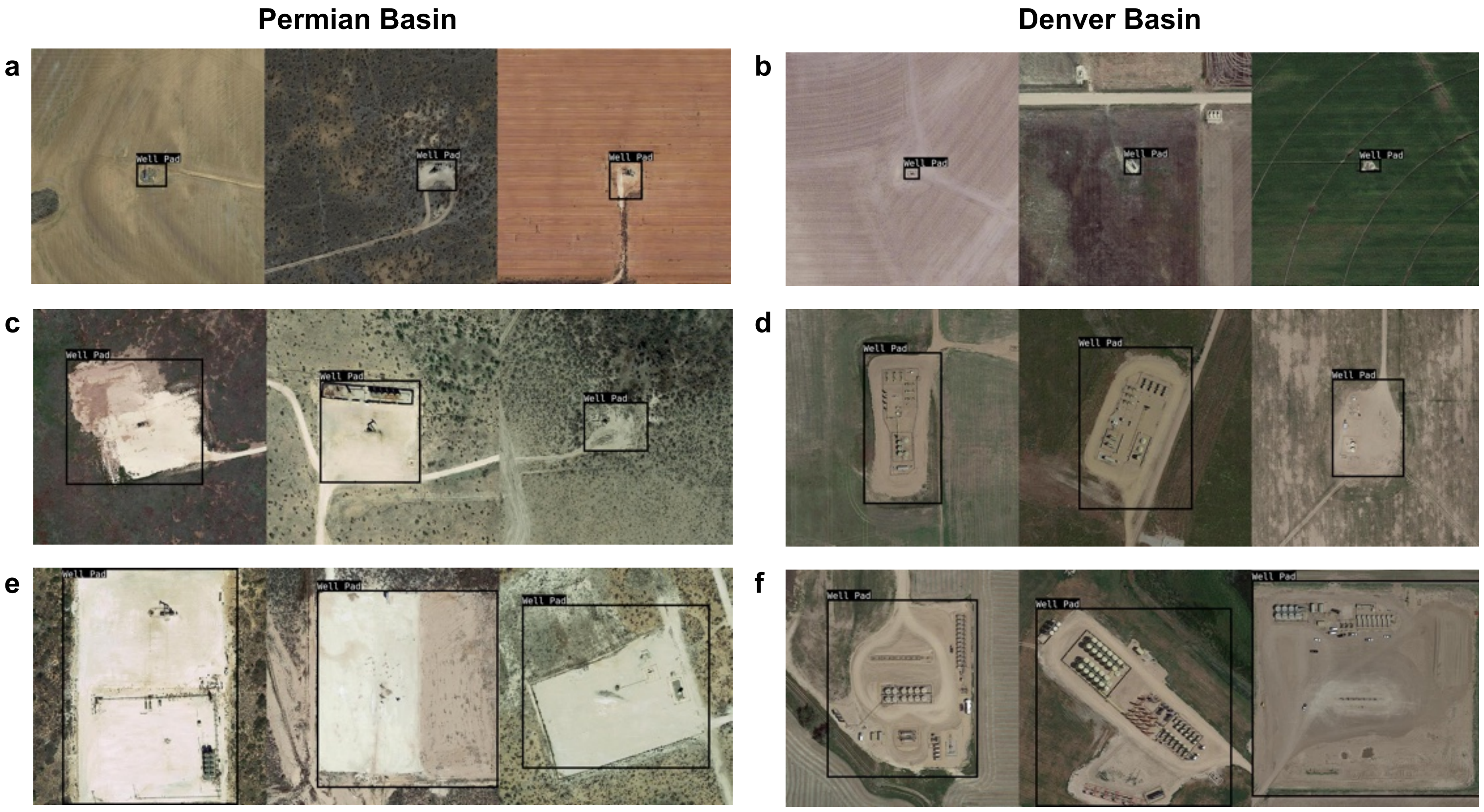

Map Data: Google, ©2013-2019

Supplementary Fig. 1. Satellite imagery of well pads. a,b Small (<41m^2), **c,d** medium (41-164m^2), and **e,f** large (>164m^2) well pads with manually obtained annotations overlaid. Performance on small well pads is considerably lower than on medium and large-sized well pads in both basins, and is poorest on small well pads in the Denver basin, where there are few discernible features on single wellhead pads and a higher likelihood of incurring false positives.

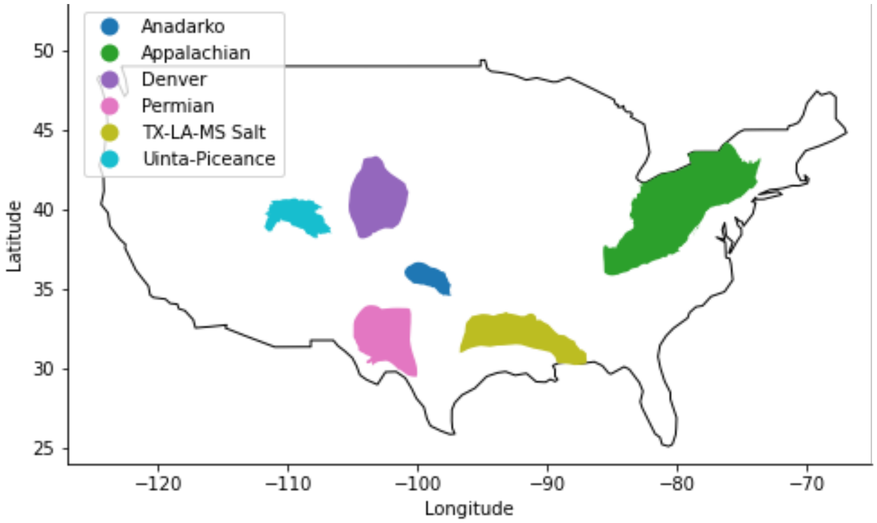

Supplementary Fig. 2: Subset of high-producing sedimentary basins in the U.S. The model was trained in the Permian and Denver basins, and evaluated in all basins shown here.

a

b

c

d

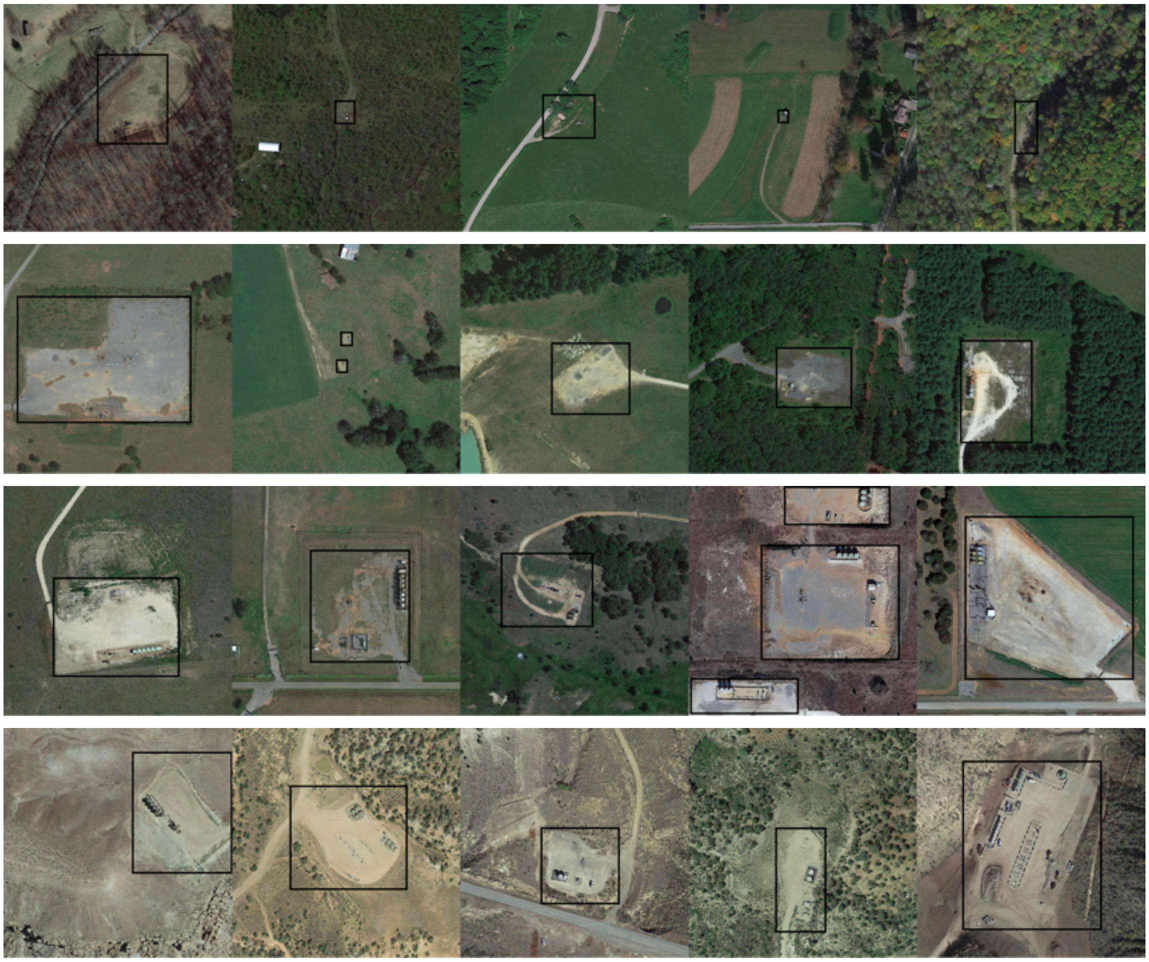

Map Data: Google, ©2013-2019

Supplementary Fig. 3. Satellite imagery of well pads in evaluation basins. The model was evaluated in the **a** Appalachian, **b** TX-LA-MS Salt, **c** Anadarko, and **d** Uinta-Piceance basins, which were unseen during training.

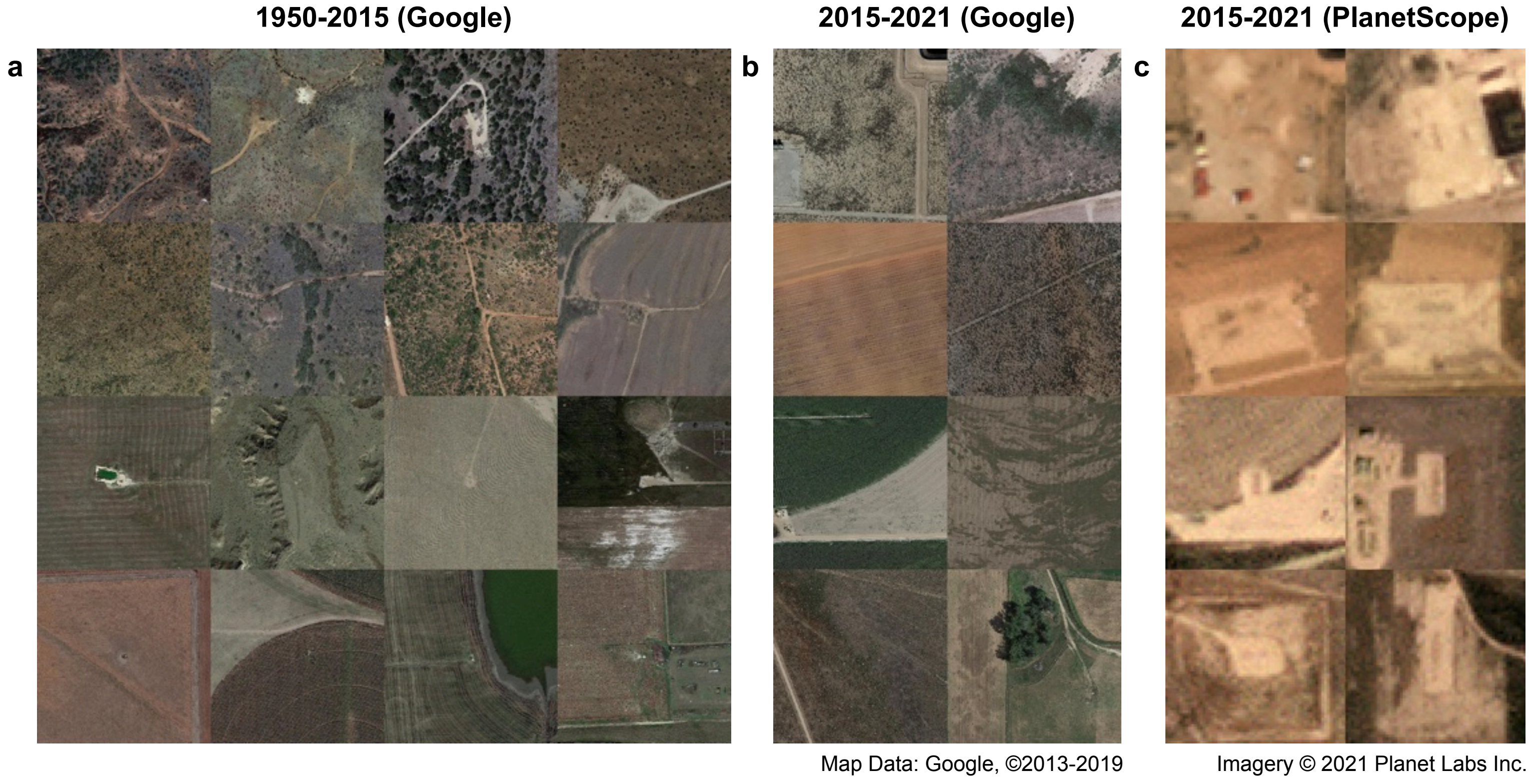

Supplementary Fig. 4. Random sample of well pad locations from the Enverus dataset that were undetected by the model. Well pads are shown from the Permian (top two rows) and Denver (bottom two rows) basins. **a** For the sample of well pads constructed from 1950-2015, the missed well pad locations primarily consist of small well pads and images with no visible well pad, indicating that the reported datasets include some data with inaccurate and/or outdated locations. **b** For the sample of well pads constructed from 2015-2021, the missed well pad locations also contain no visible well pad; however, these recent examples can be attributed to outdated imagery. **c** The same locations are shown with 2021 PlanetScope imagery, where well pads are clearly visible in the images.

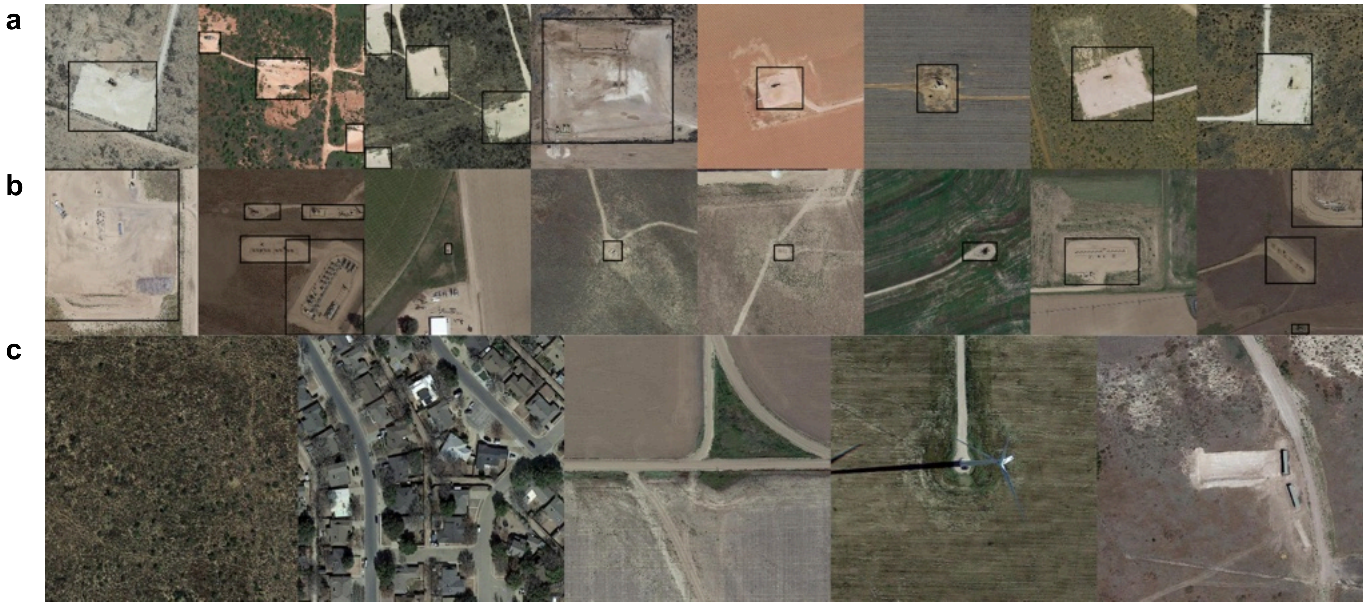

Map Data: Google, ©2013-2019

Supplementary Fig. 5. **Example satellite images in the training dataset.** Manually obtained well pad annotations used to train and evaluate the models are overlaid on the images in the **a** Permian and **b** Denver basins. **c** Example satellite images of negatives in both basins which were curated (from left to right) by random sampling within basin boundaries, within city boundaries, roads, wind turbines, and land use / land cover similar to well pads and surrounding landscapes collected using GeoVisual similarity search. Negative images are displayed larger for figure visualization but all images in the dataset are the same size.

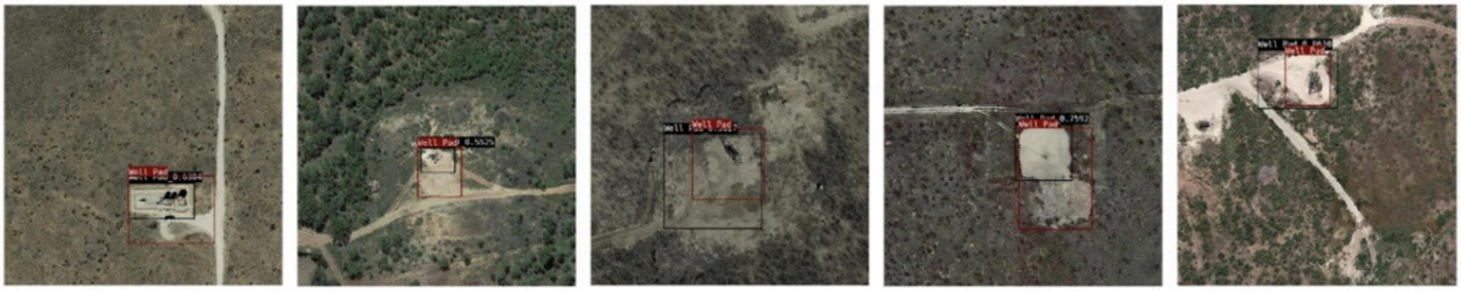

Map Data: Google, ©2013-2019

Supplementary Fig. 6. Well pads with ground truth (red) and predicted (black) bounding boxes overlaid. In each example the IoU is greater than 0.3 and less than 0.5. During model evaluation, we determined matches between predicted and ground truth bounding boxes using an IoU threshold of 0.3, a lower value than conventionally used for evaluation in object detection (which typically range from 0.5-0.95). We justified this choice based on the ambiguities in defining well pad boundaries for many well pads. The figure shows such examples, where the predictions show reasonable localizations of the well pads despite IoU < 0.5, which motivated our choice of the 0.3 IoU threshold.

a

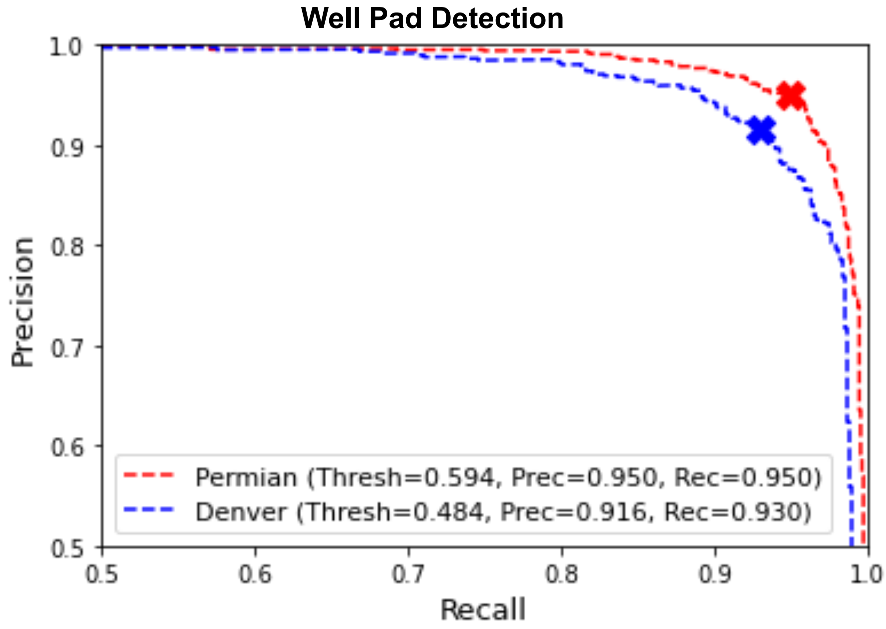

b

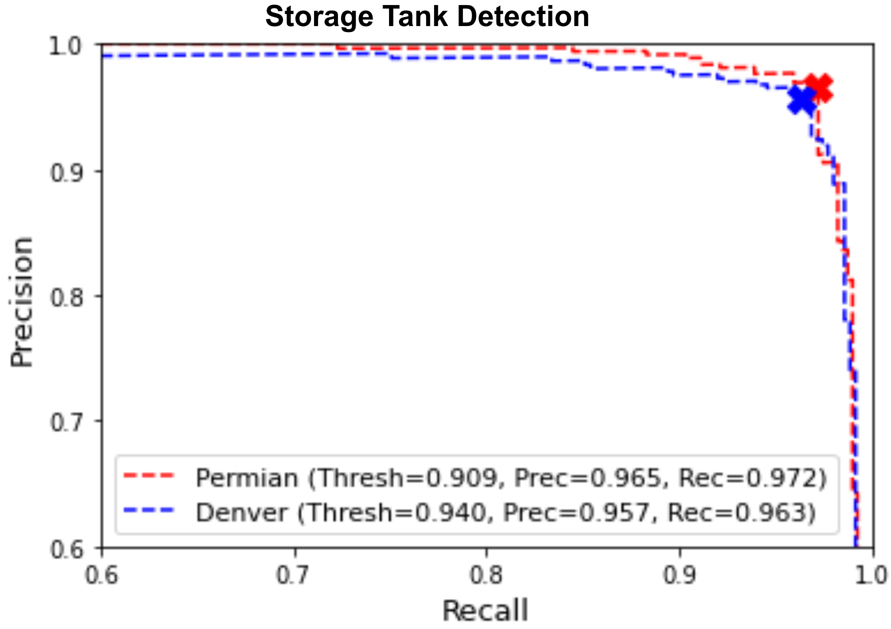

Supplementary Fig. 7: Precision-recall curve of the **a** well pad detection and **b** storage tank detection models in the Permian and Denver basins. The selected threshold value, and the corresponding precision and recall values, are shown in the legend.

Permian Basin

Denver Basin

a

b

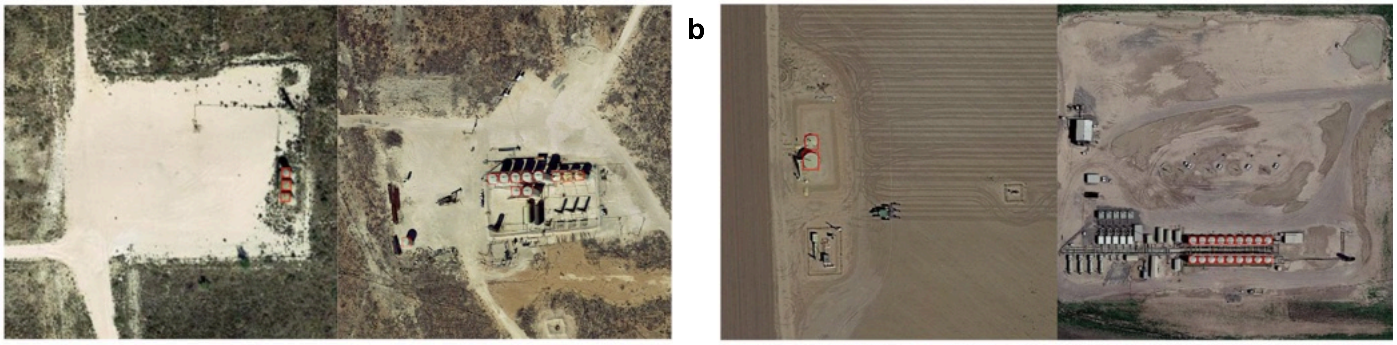

Map Data: Google, ©2013-2019

Supplementary Fig. 8. **Example satellite images of well pads with storage tanks.** Manually obtained storage tank annotation, shown in red, in the **a** Permian and **b** Denver basins were used to train and evaluate the models.

Supplementary Note 1: Construction of Well Pad Datasets

To verify and evaluate model detections during deployment, we leveraged the HIFLD and Enverus (2021 version) well datasets. To match the *well* datasets to the *well pad* model detections, we first clustered the well datasets into well pads. We used DBSCAN, a spatial clustering algorithm, with parameters min_samples set to 1 and eps set to 50m. We considered each well cluster as a well pad, and chose the centroid of well coordinates as the well pad location.

Other well-level data were also aggregated. For most categorical variables (Well Type, Production Type, Operator, etc.) the mode value in the cluster was assigned to the well pad. Dates were assigned according to the semantic meaning of the variable (ie, the oldest date was selected for First Production Date, while the most recent date was chosen for Completion Date). Production-related variables, i.e. kBOE/d (only available in the Enverus dataset) were summed for the cluster. The active/inactive status of a well pad was determined as follows: For the Enverus dataset, if the mode Well Status (“ENVWellStatus”) was “PRODUCING” or “COMPLETED” and the summed kBOE/d (“kboed”) was greater than 0, the well pad was considered active. All other well pads were considered inactive. For the HIFLD dataset, if the mode Well Status (“STATUS”) was “PRODUCING WELL” or “ACTIVE WELL”, the well pad was considered active; all other well pads were considered inactive.

The Enverus and HIFLD well databases were clustered and well pad datasets formed independently. Because the former contained some locations not present in the latter, we also formed a joint dataset constructed as the union of the locations in both datasets (Enverus + HIFLD), with duplicates within 25m removed. The joint dataset was leveraged in particular for determining which model detections could be considered “new”.

Supplementary Table 12: Number of wells in the original Enverus and HIFLD datasets, as well as the number of well pads constructed using the methodology above.

		# Wells (Original database)	# Well pads (Constructed database)
Permian	Enverus	150,542	134,039
	HIFLD	190,797	161,023
	Enverus + HIFLD	200,318	183,325
Denver	Enverus	22,148	13300
	HIFLD	30,988	24316
	Enverus + HIFLD	37,308	26285
