# Supplementary material for: Deep learning for detecting and characterizing oil and gas well pads in satellite imagery
Source: Nat Commun. 2024 Aug 15;15:7036. doi: 10.1038/s41467-024-50334-9 (PMC11327246; doi:10.1038/s41467-024-50334-9)
Supplement: Supplementary file 2 — Peer Review File [file 41467_2024_50334_MOESM2_ESM.pdf]

nature portfolio

Peer Review File

Deep learning for detecting and characterizing oil and gas well pads in satellite imagery

Open Access This file is licensed under a Creative Commons Attribution 4.0 International License, which permits use, sharing, adaptation, distribution and reproduction in any medium or format, as long as you give appropriate credit to the original author(s) and the source, provide a link to the Creative Commons license, and indicate if changes were made. In the cases where the authors are anonymous, such as is the case for the reports of anonymous peer reviewers, author attribution should be to 'Anonymous Referee' followed by a clear attribution to the source work. The images or other third party material in this file are included in the article's Creative Commons license, unless indicated otherwise in a credit line to the material. If material is not included in the article's Creative Commons license and your intended use is not permitted by statutory regulation or exceeds the permitted use, you will need to obtain permission directly from the copyright holder. To view a copy of this license, visit http://creativecommons.org/licenses/by/4.0/.

REVIEWER COMMENTS

Reviewer #1 (Remarks to the Author):

The authors propose a new framework for automatic detection of well pad and tank storage via the use of deep learning models. The detail of this framework - split into two parts: automatic detection of well pads and automatic detection of storage tanks - is clearly detailed and well organized.

One of the advantages of this work lies in the use of a binary classifier which allows eliminating false positive and verifying the detections of the deep learning model for the detection of well pads. The authors have demonstrated that adding this additional step can considerably improve the results (themselves detailed through the use of various metrics).

The results obtained via this framework also represent a clear contribution via the creation of a database which allowed the detection of 33% additional well pad and storage tank compared to other databases.

All of these results also allow us to conclude interesting and comprehensive analyses, particularly on the issues of recognition and accounting of orphan wells and the impact that the obsolescence of satellite data can have on the method.

The limits of this framework are clearly stated and the perspectives proposed seem quite insightful.

The figures presented are clear and relevant and sufficiently commented.

Remarks :

- In the central text, the transition from RetinaNet for well pad detection to that of FASTER RCNN for storage tank detection could be briefly commented on by stipulating that among a selection of modes the latter was the most efficient + possibly the addition a reference to section 4.1.2 Models for more details.

- In addition to the verification bases used, other bases such as OGIM (EDF) could perhaps have been mentioned.

Reviewer #2 (Remarks to the Author):

The authors need to demonstrate the portability of their "model," i.e., how well the trained model performs in areas other than the Permian and the Denver-Julesburg Basin.

The authors stated in the manuscript that they compared multiple models and selected the optimal model at each detection stage. However, the results of these comparisons are not provided. The authors should consider presenting the comparison results, as it will provide the reader with a clearer understanding of the basis for the selection of the models.

Lines 329-340: The authors used the detection precision obtained by the model on 500 samples to assess the total number of new well pads in the Permian and the Denver-Julesburg Basin. The authors are correct in stating that only 55,000 out of 67,201 new well pads were detected in the Permian Basin, and 15,000 out of 24,525 were detected in the Denver-Julesburg Basin. Does this indicate that the current detection precision of the method is still insufficient, leading to an inability to accurately depict the precise location of the well pads? The authors may need to provide a more detailed description of this question.

Line 515: The authors stated that the data used in this study have a spatial resolution of 30-70 m, but then state in line 481 that the 15 m spatial resolution of the Landsat 8 data is not sufficient. This statement is a contradiction in terms.

A confidence rate threshold must be determined when calculating detection precision and recall. However, this manuscript does not explicitly state the criteria for selecting this threshold. The authors should consider specifying the values for the confidence threshold and selection criteria. Furthermore, a P-R curve graph can offer a more vivid representation of the content described in lines 626-630.

Reviewer #3 (Remarks to the Author):

In this paper, the authors propose two deep learning approaches to solve two problems using images extracted from Google Earth. The first problem is the detection of well pads that is solved using a two-step approach with first a network to maximize the recall and the second to maximize the precision using the output of the first network. The second problem is the detection of storage tanks in well pads. Using the well pads detected with the first approach, this second network detects individual tanks in a pad. To train these neural networks, the authors extracted the images of Google Earth corresponding to likely positions using the approximation information of localization from the Enverus and HIFLD before manual filtering. The two approaches are then applied to larger regions of the Permian and Denver basins and statistics are compared to those of the database used to derive the training data.

In my opinion, this paper is not adapted for this journal. The work is overall interesting and has the potential of being useful but it is simply a deep learning detection methods application to well pads

and storage tank detection and therefore would fit better into more appropriate journals focusing on deep learning applications. This paper misses a clear application that could link the results to current problems cited (like methane emissions) with a case study or a global study of well pads (and storage tanks), or at least in more than the two regions used for training.

Major concerns:

- I don't think that this paper is adapted to this journal. The contribution is only two deep learning models for detections with little novelty. The models are classic and there is no clear application to the proposed pipeline. More so given that the conclusion to the study is the correlation between the detection and production is not clear and that the latest years studied (2016-2020) seems to show a drop in the quality of the results (justified by the lack of recent data from Google Earth). This means that the proposed methods cannot be used to get insight from the current O&G production, which is very important to track the emissions of greenhouse gasses and in particular methane that seems to be the focus of the study. Indeed, detecting wells two/three years later (if not more) adds little additional value to already existing methane studies using frequent and recurrent satellite imagery.

- The analysis of the generalization of the method is very limited and performed only on very similar data. For both the pad detection pipeline and the storage tank detection pipeline, the evaluation is performed on 250 examples only for each of the two basins studied! This is an extremely small sample especially when one of the presented contributions is the generalization over the entire basins studied (Permian and Denver). I don't think that extrapolating results from 250 samples to 55,000 samples makes sense. Especially since more samples could have been studied to make a more relevant study. Only looking at only two regions (especially since the two regions were used for training) is also a major limitation of the proposed study. In such a journal, I would have expected an actual general study looking at different regions of the world to try to infer interesting statistics about O&G production and not mention that it will be done in a future study.

Minor comments:

- Figure 1: the caption is split on two different pages

- Figure 2: misssed -> missed

- l. 516 "(30-70m ...)" -> I assume "(30-70cm ...)"

- It is not clear how the authors are able to distinguish between terraced regions and unregistered well pads without equipment. Indeed, these detections are referred to as new detections even though they could have been unrelated.

- A two-step approach was used to detect pads (one step to maximize the recall and the other to cleanup these detections and maximize the precision). It is curious that this approach was not also used for tank storage detection.

We thank both reviewers for their helpful comments. We have incorporated their suggestions and believe the manuscript is now substantially stronger. Our responses to the reviewers' comments are listed below, with the associated revisions in the manuscript. In summary of the major revisions to the manuscript, we:

(a) Increase the sample sizes used to estimate the performance of the well pad and storage tank models during deployment. In particular, we increase the sample size by an order of magnitude for both detection tasks, from n=500 to n=5,000 for the "new" well pad detections and from n=500 to n=10,000 for storage tank detections. For both tasks, the sample size now represents over 5% of the total detections.

(b) Evaluate the well pad and storage tank detection models' ability to generalize to new regions that were not seen during the training phase. In particular, we collect new labeled datasets in four high-producing U.S. basins, and evaluate the models in those regions.

(c) Make minor grammatical, structural, and formatting changes to comply with the Nature Communications formatting guidelines.

Our point-by-point responses to each of the reviewer comments follow below.

Reviewer 1

Reviewer Comment	Response to Reviewer	Location(s) of Edit
In the central text, the transition from RetinaNet for well pad detection to that of FASTER RCNN for storage tank detection could be briefly commented on by stipulating that among a selection of modes the latter was the most efficient + possibly the addition a reference to section 4.1.2 Models for more details.	We now briefly comment on the way we selected models for well pad detection:<br><br>“We selected the best architecture, backbone, and hyperparameters for the detection and verification models based on which led to the highest performance on the validation set (see Methods, Supplementary Tables 1-2).”<br><br>as well as for storage tank detection:<br><br>"We selected a FasterRCNN architecture with a Res2Net backbone as the highest-performing model (see Methods, Supplementary Table 7)...”<br><br>Further, we have added Supplementary Tables 1, 2, and 7, which show results of the sweep across several architectures and backbones for well pad detection, well pad verification, and storage tank detection respectively, to justify our choice of models.	Results → Training deep learning models to detect and verify well pads → Paragraph 1<br><br>Results → Storage tank detection → Paragraph 1<br><br>Supplementary Tables 1, 2, 7
In addition to the verification bases used, other bases such as OGIM (EDF) could perhaps have been mentioned.	We now include our reasoning for not using other common known bases, including OGIM:<br><br>"We considered other commonly known O&G infrastructure data repositories such as OGIM (v1.1) and GOGI (v10.3.1) but we did not use them in this study as the former sources exclusively from HIFLD in the Permian and Denver basins and the latter primarily consists of gridded well counts rather than point locations."	Methods → Deployment → Paragraph 5

Reviewer 2

Reviewer Comment	Response to Reviewer	Location(s) of Edit
The authors need to demonstrate the portability of their "model," i.e., how well the trained model performs in areas other than the Permian and the Denver-Julesburg Basin.	We thank the reviewer for the suggestion about testing the portability of the model. We now test the well pad and storage tank models in four new regions outside the Permian and Denver-Julesburg basins.<br><br>Specifically, we collected additional well pad and storage tank datasets in four U.S. basins that were unseen by the model during training and evaluated the model on these datasets. We now describe the data collection process in Methods, present the results in the central text with additional information presented in the Supplementary (due to word and figure limits), and discuss the findings in the Discussion.<br><br>We note that we do not deploy the model at the basin-scale in these new regions, as including those analyses would significantly change the scope and presentation of our work.	Methods → Training dataset for well pads → Paragraph 8;<br>Methods → Storage tank detection → Paragraph 1<br><br>Results → Training deep learning models to detect and verify well pads → Paragraph 7;<br>Results → Storage Tank Detection → Paragraph 4<br><br>Supplementary Tables 5,8;<br>Supplementary Figs 2,3<br><br>Discussion → Paragraph 2

The authors stated in the manuscript that they compared multiple models and selected the optimal model at each detection stage. However, the results of these comparisons are not provided. The authors should consider presenting the comparison results, as it will provide the reader with a clearer understanding of the basis for the selection of the models.	As suggested by the reviewer, we have added the results of the model comparisons to the Supplementary which we now refer to in the main text for well pads:<br><br>“We selected the best architecture, backbone, and hyperparameters for the detection and verification models based on which led to the highest performance on the validation set (see Methods, Supplementary Tables 1-2).”<br><br>and storage tank detection:<br><br>"We selected a FasterRCNN architecture with a Res2Net backbone as the highest-performing model (see Methods, Supplementary Table 7)...”	Supplementary Tables 1, 2, 7<br><br>Results → Training deep learning models to detect and verify well pads → Paragraph 1<br><br>Results → Storage tank detection → Paragraph 1
Lines 329-340: The authors used the detection precision obtained by the model on 500 samples to assess the total number of new well pads in the Permian and the Denver-Julesburg Basin. The authors are correct in stating that only 55,000 out of 67,201 new well pads were detected in the Permian Basin, and 15,000 out of 24,525 were detected in the Denver-Julesburg Basin. Does this indicate that the current detection precision of the method is still insufficient, leading to an inability to accurately depict the precise location of the well pads? The authors may need to provide a more detailed description of this question.	We first note that we have re-evaluated the "new" detections on a sample size an order of magnitude larger than previously (n=5,000). We find rates of correctly detected well pads in the new sample, leading to new estimates of 55,800/67,201 (83.0%) and 14,200/24,525 (57.9%) actual well pads among the new detections in the Permian and Denver basins, respectively.<br><br>The reviewer suggests that these numbers may imply that the precision of the method is insufficient, which we argue is not the case. We first note that the numbers provided above only assess "new" detections, i.e. detections that did not match well pads in the union of the HIFLD and Enverus datasets, and do not factor in the number of "captured" detections, i.e. detections that did match the reported datasets. In particular, when including captured detections, 190,547 out of 201,948 (94.4%) detections in the Permian and 26,528 out of 36,853 (72.0%) detections in the Denver basin are	N/A

	actual well pads, which are the precision estimates we report in Section 2.2. We argue that these precision levels are sufficient, as if a higher precision is desired, the model can be viewed as an effective way to propose well pad detections that can be validated by human review. For example, precision can be improved to 100% by performing a human review of the 91,726 new detections in both basins. We note that the precision of the model is sufficient to make such an effort tractable (for reference, we reviewed n=2,500 samples in the basin in approximately 3 hours, which scales to approximately 110 hours for all new detections), whereas if the precision of the model were lower such an effort would be impractical.<br><br>Finally, we acknowledge that the precision values estimated from the basin-scale deployment are lower than the precision of the model evaluated on our test dataset, which we also comment on at length, including how future work can address this, in the Discussion section.	
Line 515: The authors stated that the data used in this study have a spatial resolution of 30-70 m, but then state in line 481 that the 15 m spatial resolution of the Landsat 8 data is not sufficient. This statement is a contradiction in terms.	We thank the reviewer for pointing out this error. This line should have read "30-70cm", which then does not contradict the Landsat resolution. We have corrected this typo.	Location specified by the Reviewer.
A confidence rate threshold must be determined when calculating detection precision and recall. However, this manuscript does not explicitly state the criteria for selecting this threshold. The authors should consider specifying the values for the confidence threshold and selection criteria. Furthermore, a P-R curve graph can offer a more vivid representation of the content described in lines 626-630.	We thank the reviewer for their suggestion, which helps provide clarity about the precision-recall tradeoff and our choice of threshold.<br><br>We do state the criteria for our choice of threshold in the Methods section: "[W]e specifically measured performance on the validation set at thresholds corresponding to 95% recall in the Permian basin and	 <br><br><br>Methods → Model training and evaluation → Paragraph 9

	93% recall in the Denver basin in order to increase the completeness of the dataset when the model is deployed."<br><br>However, we did not explicitly specify the threshold values as suggested by the reviewer. We address this suggestion, along with the addition of a P-R curve by adding a figure to the Supplementary and briefly refer to the figure in the Methods section.	 <br><br><br>Supplementary Fig. 7<br><br>Methods → Model training and evaluation → Paragraph 9

Reviewer 3

Reviewer Comment	Response to Reviewer	Location(s) of Edit
In my opinion, this paper is not adapted for this journal. The work is overall interesting and has the potential of being useful but it is simply a deep learning detection methods application to well pads and storage tank detection and therefore would fit better into more appropriate journals focusing on deep learning applications. This paper misses a clear application that could link the results to current problems cited (like methane emissions) with a case study or a global study of well pads (and storage tanks), or at least in more than the two regions used for training.	We first highlight that we have now evaluated the approach in four more high oil and gas producing regions beyond the two regions used for training. We agree with the reviewer that linking the data to methane emissions is an interesting follow-up analysis, among several others that we describe in the Discussion, but we leave this to future work which uses the data we have created.<br><br>We appreciate the reviewers comments about journal fit, but respectfully disagree. Several similar application-focused deep learning works have been published in Nature Communications recently (Wu et al., 2023; Yeh et al., 2020; Guirado et al., 2019). We believe the effectiveness, scalability, and impact of our work to address an important and urgent challenge makes it a good fit for this journal.	N/A
I don't think that this paper is adapted to this journal. The contribution is only two deep learning models for detections with little novelty. The models are classic and there is no clear application to the proposed pipeline. More so given that the conclusion to the study is the correlation between the detection and production is not clear and that the latest years studied (2016-2020) seems to show a drop in the quality of the results (justified by the lack of recent data from Google Earth). This means that the proposed methods cannot be used to get insight from the current O&G production, which is very important to track the emissions of greenhouse gasses and in particular methane that seems to be the	Our main goal was to develop an effective and scalable approach for detecting well pads, and found that existing methods, with careful design and data curation, can perform this task successfully. As we state at the end of the introduction, our main contribution is not technical novelty, but the dataset curation, rigorous experiments, and construction of a publicly available database of hundreds of thousands of well pad locations (filling data gaps in existing public and private databases) and storage tank locations (which did not previously exist).<br><br>We agree that a limitation of the approach is the lack of	

focus of the study. Indeed, detecting wells two/three years later (if not more) adds little additional value to already existing methane studies using frequent and recurrent satellite imagery.	recent imagery, which limits our ability to detect new, high-producing well pads and is one of the key findings of our work. This will help inform future studies working on identifying oil and gas infrastructure using AI on satellite imagery. Nevertheless, improved mapping of older well pads is still crucial, which we now defend in the Discussion:<br><br>"Despite this limitation, our ability to fill gaps in the mapping of older, lower-producing well pads is significant, as previous work showed that such well pads account for a disproportionately large amount of methane emissions (Omara et al., 2022)"<br><br>Thus, the ability to detect slightly older well pads, and to fill gaps in the historical datasets, is valuable for improving our understanding of methane emissions, i.e. through better source attribution and bottom-up estimates.	 <br><br><br><br><br><br><br>Discussion, Paragraph 10
The analysis of the generalization of the method is very limited and performed only on very similar data. For both the pad detection pipeline and the storage tank detection pipeline, the evaluation is performed on 250 examples only for each of the two basins studied! This is an extremely small sample especially when one of the presented contributions is the generalization over the entire basins studied (Permian and Denver). I don't think that extrapolating results from 250 samples to 55,000 samples makes sense. Especially since more samples could have been studied to make a more relevant study. Only looking at only two regions (especially since the two regions were used for training) is also a major limitation of the proposed study. In such a journal, I would have expected an actual general study looking at different regions of the world to try to infer interesting	We thank the reviewer for pointing out this limitation of the study, and acknowledge that the sample sizes for the human-evaluated well pad and storage tank detections were small relative to the total number of detections.<br><br>To address this limitation, we re-evaluate the detections, increasing both sample sizes by an order of magnitude. We now sample n=5,000 new well pad detections (n=2,500 in each basin), which comprise over 5% of the total new detections. We also now sample n=10,000 storage tank detections (n=5,000 in each basin), which comprise over 5% of the total storage tank detections. We update any relevant estimates in the paper that were calculated based on the sample.	 <br><br><br><br><br>Results → Basin-scale well pad deployment → Paragraphs 8-9<br><br><br>Results → Storage tank detection → Paragraph 6

statistics about O&G production and not mention that it will be done in a future study.	We address the reviewer's comment about the limited analysis of the generalization of the method in the next comment.	
Only looking at only two regions (especially since the two regions were used for training) is also a major limitation of the proposed study. In such a journal, I would have expected an actual general study looking at different regions of the world to try to infer interesting statistics about O&G production and not mention that it will be done in a future study.	We thank the reviewer for the suggestion about expanding the scope of the study beyond the regions used for training the model. We have now evaluated the approach for well pads and storage tanks in four more high oil and gas producing regions beyond the two regions used for training.<br><br>Specifically, we collected additional well pad and storage tank datasets in four U.S. basins that were unseen by the model during training and evaluated the model on these well pad datasets. We now describe the data collection process in Methods, present the results in the central text with additional information presented in the Supplementary (due to word and figure limits), and discuss the findings in the Discussion.<br><br>We note that we do not deploy the model at the basin-scale in these new regions, as including those analyses would significantly change the scope and presentation of our work.	Methods → Training dataset for well pads → Paragraph 8; Methods → Storage tank detection → Paragraph 1<br><br>Results → Training deep learning models to detect and verify well pads → Paragraph 7;<br>Results → Storage Tank Detection → Paragraph 4<br><br>Supplementary Tables 5,8; Supplementary Figs 2,3<br><br>Discussion → Paragraph 2
Figure 1: the caption is split on two different pages	We have now fit the caption on a single page.	Location specified by reviewer
Figure 2: misssed -> missed	We have fixed this typo.	Location specified by reviewer
l. 516 "(30-70m ...)" -> I assume "(30-70cm ...)"	We have fixed this typo.	Location specified by reviewer
It is not clear how the authors are able to distinguish between terraced regions and unregistered well pads without equipment. Indeed, these detections are	The reviewer raises a valid point concerning our ability to discern between "terraced regions" and unregistered well pads without equipment. During our evaluation of	

referred to as new detections even though they could have been unrelated.	the new detections, we categorized 21.6% of the detections as bare well pads, i.e. "completely bare, i.e. containing no visible equipment such as pump jacks, storage tanks, wellhead fencing, or wellheads." We note that bare well pads may still be methane emitters as they may be plugged and abandoned/orphaned well pads, or footprints that have been cleared but not yet drilled at the time of imagery acquisition.<br><br>However, the reviewer implies that the detections we classify as bare well pads could also be regions terraced for some unrelated purpose. While this is possible, we take into account features beyond just visible equipment when making the classification, which we have now clarified in the paper:<br><br>“We also note that we distinguish the bare “well pads” from land cleared for other purposes (e.g. agriculture) through features such as proximity to other well pads, presence of characteristic road(s) leading to the site, and proximity to other infrastructure, which often indicate that a cleared area is not a well pad (i.e. a cleared region next to a farm is unlikely to be a well pad and more likely to be associated with agricultural use).”	Results → Basin-scale well pad deployment → Paragraph 9
A two-step approach was used to detect pads (one step to maximize the recall and the other to cleanup these detections and maximize the precision). It is curious that this approach was not also used for tank storage detection.	We do not adopt the two-step approach for detecting storage tanks for two primary reasons, which we have added to the paper:<br><br>“We do not adopt the two-stage approach we used previously for detecting storage tanks because (a) the detection model achieves high precision and recall on its own and (b) verifying individual instances of storage tanks is difficult, as they often appear in clusters or in close proximity.”	Results → Storage tank detection → Paragraph 1

REVIEWERS' COMMENTS

Reviewer #2 (Remarks to the Author):

The authors have addressed my comments and suggestions that I proposed in the first round of review. As a result, I recommend the revised manuscript for publication.

Reviewer #3 (Remarks to the Author):

The authors have answered all issues raised by the different reviewers. While there are still points that would benefit from additional details (for example, how features are used to discriminate between well pads and other structures or the performance of a two stage detector for the storage tanks), these are not major and therefore I don't have any issue with the paper being published.

Reviewer #3 (Remarks on code availability):

If I'm not mistaken, training scripts are not provided with the code. Also, while there is a README provided, it doesn't contain examples on how to run training and/or eval for the provided code.

We thank the reviewers for their helpful comments. Our point-by-point responses to Reviewer #3's remarks are below..

Reviewer 3

Reviewer Comment	Response to Reviewer	Location(s) of Edit
The authors have answered all issues raised by the different reviewers. While there are still points that would benefit from additional details (for example, how features are used to discriminate between well pads and other structures or the performance of a two stage detector for the storage tanks), these are not major and therefore I don't have any issue with the paper being published.	We thank the reviewer for the multiple rounds of review.<br><br>We note to the reviewer that we added details related to how features are used to discriminate between well pads and other structures in the last round of review:<br><br>"We note that 21.6% of new well pad detections in the sample were completely bare, i.e. containing no visible equipment such as pump jacks, storage tanks, well head fencing, or well heads (which may in some cases be too small to see in satellite imagery) typically used to discern well pads from other infrastructure…We also note that we distinguish the bare "well pads" from land cleared for other purposes (e.g. agriculture) through features such as proximity to other well pads, presence of characteristic road(s) leading to the site, and proximity to other infrastructure, which often indicate that a cleared area is not a well pad (i.e. a cleared region next to a farm is unlikely to be a well pad and more likely to be associated with agricultural use)."<br><br>In regards to the performance of a two stage detection pipeline for storage tanks, we also added details in the previous round of review about why such a pipeline worked naturally for well pads but not for storage tanks:<br><br>"We do not adopt the two-stage approach we used previously for detecting storage tanks because (a) the detection model achieves high precision and recall on	Results → Basin-scale well pad deployment → Paragraph 9<br><br>Results → Storage tank detection → Paragraph 1

	its own and (b) verifying individual instances of storage tanks is difficult, as they often appear in clusters or in close proximity."	
If I'm not mistaken, training scripts are not provided with the code. Also, while there is a README provided, it doesn't contain examples on how to run training and/or eval for the provided code.	The reviewer is correct that training scripts are not provided in the code repo. We note that although we have provided coordinate locations of the well pads and storage tanks in the training datasets, we are unable to release the imagery the models were trained on and thus the training code cannot be run. As such, we have opted not to release the training code and have only released the evaluation code for replicating results in the paper. We note that the training code primarily makes use of popular deep learning libraries mmdetection and pytorch-lightning for training the models.<br><br>We have clarified the above in the Data Availability/Code Availability statements, while also noting that the training scripts may be shared with readers upon request.<br><br>In the README of the code repo, we have also clarified how to run the evaluation scripts for the provided code, in regards to the reviewer's second remark.	Data Availability/Code Availability Statements in main manuscript;<br><br>README in CodeOcean repo
